# Evaluating the Effects of Corn Flour Product Consumption on Cardiometabolic Outcomes and the Gut Microbiota in Adults with Elevated Cholesterol: A Randomized Crossover

**DOI:** 10.1016/j.tjnut.2024.06.003

**Published:** 2024-06-14

**Authors:** Bethany Liedike, Maissa Khatib, Baharak Tabarsi, Michelle Harris, Shannon L Wilson, Carmen P Ortega-Santos, Alex E Mohr, Sonia Vega-López, Corrie M Whisner

**Affiliations:** 1 College of Health Solutions, Arizona State University, Phoenix, AZ, United States; 2 Community Health Center, Valleywise Health, Phoenix, AZ, United States; 3 Department of Exercise and Nutrition Sciences, Milken Institute School of Public Health, The George Washington University, Washington, DC, United States; 4 Center for Health Through Microbiomes, The Biodesign Institute, Arizona State University, Tempe, AZ, United States; 5 Southwest Interdisciplinary Research Center, Arizona State University, Phoenix, AZ, United States

**Keywords:** cholesterol, corn flour, gut microbiota, dietary fiber, whole grain

## Abstract

**Background:**

Consumption of whole grains is associated with a reduction in chronic diseases and offers benefits for cardiovascular health and metabolic regulation. The relationship between whole-grain corn and corn bran with the gut microbiota (GM) remains an area of growing interest, particularly regarding their influence on cardiometabolic health.

**Objectives:**

To investigate the effects of different corn flours on cardiometabolic outcomes and GM changes in adults with elevated low-density lipoprotein cholesterol (LDL cholesterol) concentrations.

**Methods:**

In this crossover study, 36 adults with LDL cholesterol above 110 mg/dL consumed 48 g/d of 3 corn flour types for 4 wk: whole-grain corn meal, refined corn meal (RCM), and a blend of RCM and corn bran (RCM + B). We assessed the impact on cardiometabolic markers [LDL cholesterol, high-density lipoprotein cholesterol (HDL cholesterol), total cholesterol, and triglycerides)] and GM composition and estimated function. Statistical analyses included mixed-effects modeling and responder (>5% decrease in LDL cholesterol) analysis to evaluate changes in GM related to lipid profile improvements.

**Results:**

Of the 3 corn flour types, only RCM + B significantly decreased LDL cholesterol over time (–10.4 ± 3.6 mg/dL, *P* = 0.005) and marginally decreased total cholesterol (–9.2 ± 3.9 mg/dL, *P* = 0.072) over time. There were no significant effects on HDL cholesterol or triglyceride concentrations. No significant changes were observed in GM alpha diversity, whereas beta diversity metrics indicated individual variability. Two genera, unclassified *Lachnospiraceae* and *Agathobaculum* (*P*_adj_ ≤ 0.096), differed significantly by treatment, but only *Agathobaculum* remained significantly elevated in the whole-grain corn meal, compared to RCM and RCM + B, after adjustment for multiple comparisons.

**Conclusions:**

The type of corn flour, particularly RCM + B, notably influenced LDL cholesterol concentrations in adults with elevated LDL cholesterol. This study suggests that incorporating milled fractions (e.g., bran) of whole-grain corn with refined corn flour may be a viable alternative to supplementing manufactured grain products with isolated or synthetic fibers for improved metabolic health.

This trial was registered at clinicaltrials.gov as NCT03967990.

## Introduction

Evidence suggests that whole grain consumption lowers the risk of chronic diseases such as cardiovascular disease [1], diabetes [2], and cancer [3]. The beneficial effects of whole grains are often attributed to their dietary fiber content [[4], [5], [6]] and associated bioactive compounds, such as polyphenols [7]. However, the specific mechanisms underlying these health benefits remain to be fully elucidated. To date, the majority of research has explored wheat-based foods and isolated fibers, leaving the influence of habitual fiber consumption from other grains less well-established [6,8].

Corn has a fiber content similar to that of wheat, with insoluble fiber comprising the largest fraction [9]. Although the insoluble fiber content of corn is primarily cellulose and hemicellulose, it also contains the health-promoting bioactives arabinoxylan and ferulic acid [10]. Of the prior studies of corn as a cereal grain, all have been of adults aged 18–70 y (2 studies included adults with hypercholesterolemia) aiming to evaluate the impact of corn on blood lipids in combination with other dietary interventions, including low-fat diets [11], other grain-based foods [12], and fiber supplements [13]. Consumption of whole-grain corn combined with whole-grain wheat and rice products, providing ∼14 g/d of fiber per 2000 kcal/d, resulted in significant reductions in LDL cholesterol and non-HDL cholesterol when compared to refined grain products among non-whole-grain consuming healthy adults [12]. Two studies investigating the effects of 20 g/d of fiber (exclusively as corn bran + a low-fat diet or corn bran combined with other dietary fibers) for 6 [11] and 51 wk [13], respectively, resulted in significant reductions in LDL cholesterol in males and females with hypercholesterolemia. Although cereal fibers have been shown to be particularly effective in reducing cardiovascular disease risk [14], the specific components of cereal grains (e.g., germ, bran, polyphenols, etc.) responsible for these effects remain underexplored.

Recent research has emphasized the crucial role of gut microbiota (GM) in health, with controlled studies linking gut microbial community structure to improved inflammatory responses via isolated dietary fibers [15]. Consumption of 48 g/d of whole-grain corn cereal for 21 d was effective at enhancing *Bifidobacterium* content [16], whereas extracted corn bran arabinoxylans at 25 g/d and 35 g/d for 6 wk among adult females and males, respectively, altered the diversity of GM while promoting the abundance of *Bifidobacterium longum*, *Blautia obeum*, and *Prevotella copri*, all dietary fiber fermenters [17]. Although dietary fibers, both natural and synthetic, have been shown to promote the growth of beneficial microbial species in the gastrointestinal tract and are associated with improvements in inflammatory and metabolic disease markers [18,19], corn remains understudied. Further, no studies have explored interactions between GM and cardiovascular health following corn consumption.

The existing, albeit limited, literature supports the health benefits of corn but is insufficient for understanding the unique roles of whole-grain corn and grain components (e.g., corn bran) in cardiometabolic health. Whole-grain corn meal (WCM) includes all parts of the grain, providing a rich source of dietary fiber and phytonutrients, whereas refined corn meal (RCM) with the bran and germ removed results in highly palatable products with lower fiber content and potentially reduced health benefits. However, the addition of corn bran to RCM (RCM + B) may combine the palatability and manufacturing benefits (e.g., longer shelf life) of refined corn with the health benefits of fiber-rich bran. Therefore, the purpose of this study was to investigate the effects of 3 different corn flours (48 g/d) for 4 wk: WCM, RCM, and a ∼70:30 blend of RCM and corn bran (RCM + B) on cardiometabolic outcomes [LDL cholesterol, HDL cholesterol, total cholesterol (TC), and triglycerides (TG)] and GM changes in adults with mild-to-moderately elevated LDL cholesterol concentrations (>110 mg/dL). An exploratory objective entailed performing a responder analysis that assessed changes within the GM among participants with improved lipid profiles.

## Methods

### Study participants and screening

The study enrolled healthy male and female participants aged 18–70 y, specifically targeting individuals with mild-to-moderately elevated LDL cholesterol (LDL cholesterol ≥110 mg/dL). The minimum LDL cholesterol concentration to qualify for the study was originally set at ≥120 mg/dL but was decreased halfway through the study to enhance enrollment of individuals not receiving statin drugs. These classifications were selected to slightly exceed the National Cholesterol Education Program Adult Treatment Panel III standards, indicating that an LDL cholesterol <100 mg/dL is optimal [20]. Individuals with LDL cholesterol >190 mg/dL were asked to provide a letter from their physician confirming their suitability for participation in the study. Participants were excluded if they had experienced significant weight fluctuations (>2.3 kg) in the previous 3 mo or if they were following specialized or restrictive diets, such as carbohydrate-restricted or vegan diets. Additionally, individuals taking supplements (antioxidants, fiber, and botanicals), those with allergies to dairy, egg, wheat, corn, or gluten, and those who had used antibiotics in the past 3 mo were excluded. The study also excluded individuals using lipid-lowering medications, those engaging in regular physical activity (defined as ≥30 min/d for ≥5 d/wk), and those with a history of thyroid disorders, diabetes, heart disease, cancer, hepatitis, inflammatory conditions, and gastrointestinal disorders that could affect gut function and metabolism. Further, female participants who were pregnant or lactating were not eligible for the study. Finally, willingness to comply with all study protocols was a prerequisite for participation. This study was approved by the institutional review board of Arizona State University (IRB#: STUDY00007518) and registered at clinicaltrials.gov (ID#: NCT03967990). Written informed consent was obtained from each participant prior to the commencement of the study.

Potential participants completed an online prescreening and interest questionnaire with ≤37 questions via Qualtrics XM Platform (Qualtrics, LLC), ensuring anonymity by deactivating the collection of Internet Protocol (IP) addresses. Qualified individuals were then contacted by study personnel via e-mail or phone (provided at the end of the questionnaire) to schedule the first visit and receive instructions for the next steps. After verbal or e-mail consent, participants arrived at the laboratory after a 12 h overnight fast for a blood draw screening. The screening consent form, explaining the procedures of the blood draw, was signed and dated by participants and investigators. A trained nurse or phlebotomist drew ∼7 mL of blood to verify the presence of mild-moderately high blood cholesterol (LDL cholesterol ≥ 110 mg/dL). After this, a general health and demographic questionnaire was filled out, and personal contact information was collected. Participants with confirmed elevated cholesterol concentrations (LDL cholesterol ≥ 110 mg/dL) were then provided with a final informed consent form to enroll in the study, along with a stool sample collection kit for baseline fecal sample collection prior to their baseline visit. Additionally, participants completed a 3-d (1 weekend day and 2 weekdays) diet record to characterize habitual dietary intake using booklets for self-report that included guidance for portion sizes and reporting details that are often forgotten (e.g., brands, cooking methods, beverages, condiments, etc.).

### Study design and dietary intervention

This study was conducted in Phoenix, AZ, as a randomized, single-blinded, 3x3 crossover study, where each participant served as their own control. Randomization was achieved by drawing from a hat so that equal-sized sequence lots would be achieved. Each intervention phase lasted 4 wk, separated by 2-wk washout periods between the 3 corn flour interventions. These time intervals are supported by the literature to allow enough time to observe dietary effects on both blood lipids [21] and the GM [[22], [23], [24]] while also allowing for an adequate washout to baseline levels. Participants consumed, in randomly assigned order, 48 g/d of 1 of 3 corn flour types: *1*) WCM, *2*) RCM + B, or *3*) RCM. The 48 g/d intake was split into 2 24 g servings, consumed either with meals or as snacks and spaced ≥3 h apart. The corn flours were provided in supplemental food items (muffins and pita bread) and were incorporated into participants’ habitual diets as replacements for wheat, oats, and other grain-rich products. Participants received 10 pita breads and 4 muffins per week. Fewer muffins were provided due to their higher caloric content (pita ∼163 kcal/serving; muffin ∼308 kcal/serving). Participants were asked to spread muffin consumption across the week and avoid eating 2 muffins per day.

After eligibility verification and before the beginning of the first intervention, participants underwent a baseline fasting (12 h) blood draw of 30 mL to complete a biomarker panel and archive samples for future analyses. Baseline questionnaires on gastrointestinal symptoms were completed. Measurements of height, weight, and waist circumference were taken 3 times and the mean was retained as a final value. Lastly, participants provided a fecal sample for GM assessment. Participants received a 1-wk supply of corn-based foods, consumption instructions, and a weekly compliance calendar. Weekly, participants received replenished study food supplies either by home delivery or pick-up. Before the end of the 4-wk intervention period, participants were given a new fecal sample collection kit. Participants also completed weekly gastrointestinal symptom surveys and food acceptability and satisfaction surveys, either online or in paper form. At the end of each 4 wk intervention period, participants attended another visit where a fasting (12 h) blood draw of 30 mL was conducted, and week 4 intervention questionnaires were completed. Participants returned uneaten foods and completed compliance calendars and a post-intervention fecal sample was either scheduled for pick-up or brought to the visit. Another fecal sample collection kit was provided for baseline collection before the next intervention phase. Following 2-wk washout periods, participants began their second and third corn flour treatments, respectively, adhering to the same protocols as described above.

### Food products

In collaboration with a master baker familiar with common food formulation techniques and specialty ingredients used in the manufacturing and production of mass-produced consumer-based foods, muffin and pita bread recipes were developed to deliver 48 g/d of each corn flour in 2 servings. Ingredients and recipes for the food products given for each treatment are presented in Supplemental Table 1 and the nutritional data in Supplemental Table 2. To produce the RCM + B flour blend, RCM was thoroughly mixed with finely milled corn bran (80% dietary fiber by weight as measured by QD230/AOAC 991.43 (Eurofins Scientific). Given the amount of fiber in the bran, a total of 7.5 g of corn bran was required for each 16.5 g of RCM to achieve a 24 g serving (total daily intake of 48 g) of corn in each pita or muffin. This roughly 30:70 ratio ensured a dietary fiber content per muffin/pita of ≥6 g to emulate consumer products worthy of a Food and Drug Administration-approved “excellent fiber source” health claim.

### Compliance, study food acceptability, and gastrointestinal symptoms

Compliance was determined by the following equation: compliance = (number of food items consumed/number of food items expected to be consumed) × 100%. Participants with >80% compliance were included in the analysis. A 3-d dietary record was conducted during each intervention to monitor diet consistency. Self-reported dietary intake was guided with a paper food record that included instructions for reporting eating episodes, portions, and commonly forgotten items (e.g., water, condiments, cooking methods, etc.). Completed food records were entered into the Nutritional Data System for Research software [25] by trained staff nutritionists to understand participants’ mean habitual intake. Participants were contacted weekly to follow up on regular food item ingestion, to inform them of treatment changes, and to note adverse effects that may have arisen subsequent to the treatment. In addition, participants completed weekly gastrointestinal symptom surveys and food acceptability and satisfaction surveys, either online or in paper form.

### Clinical and biochemical measurements

Fasting blood samples were collected prior to and at the end of each dietary intervention period. Fasting plasma lipids (TC, HDL cholesterol, and TG) were measured in duplicate with an automated chemistry analyzer (AU480 Chemistry Analyzer; Beckman Coulter) using colorimetric enzymatic reagents. LDL cholesterol was calculated as described by Friedewald et al. [26].

### Fecal sample collection and microbiota sequencing

Study participants collected whole fecal samples (entire bowel movement) prior to and at the end of each corn flour treatment using a stool sample collection kit (Commode Specimen Collection Kit, Fisher Scientific). Kits included a toilet frame and sample storage bowl and lid, in addition to zip-seal bags for double-containment of the sample storage container. Ice packs and insulated coolers were provided to ensure that the sample was kept cold until the study staff retrieved the sample. Participants were instructed to collect samples on the day of study visits or to contact study staff directly after collection so that the sample could be picked up by study staff and frozen at –80°C within 24 h of collection. Samples were processed after defrosting at 4°C by first placing them into a sterile bag and manually homogenizing them to ensure a more representative mixture of microbes within the feces. Approximately 0.25 g of the homogenized feces was transferred to PowerBead Pro tubes from the DNeasy PowerSoil Pro Kit (Qiagen).

DNeasy PowerSoil Pro Kits were used to extract microbial DNA from each fecal sample using the manufacturer protocol with no amendments. In brief, these kits use ethanol and salt-based reagents, combined with centrifugal filtration, to remove fecal matter and other inhibitors and separate microbial DNA following an initial bead-beating step that results in the lysis of microbial cell membranes and DNA release.

Amplification of the 16S rRNA gene sequence was completed in triplicate polymerase chain reactions using 96-well plates. Barcoded universal forward 515F primers and 806R reverse primers containing Illumina adapter sequences, which target the highly conserved V4 region, were used to amplify microbial DNA [27,28]. Polymerase chain reaction, amplicon cleaning, and quantification were performed as previously outlined [28]. Equimolar ratios of amplicons from individual samples were pooled together before sequencing on the Illumina platform (Illumina MiSeq instrument; Illumina, Inc.). Raw Illumina microbial data were cleaned by removing short and long sequences, sequences with primer mismatches, uncorrectable barcodes, and ambiguous bases using the Quantitative Insights into Microbial Ecology 2 software, version 2023.9 [29].

Paired-end, demultiplexed data were imported and analyzed using Quantitative Insights into Microbial Ecology 2 software. By examining sequence quality, the base pairs were trimmed at position 20, truncated at position 240, and processed through DADA2 (version 1.26.0) to remove low-quality regions and construct a feature table using amplicon sequence variants (ASVs). The maximum expected errors were set to 2 for both forward (max_ee_f) and reverse reads (max_ee_r). Reads were truncated at the first position, where the quality score (Q value) dropped below 2 (trunc_q). An independent pooling method (pooling_method) was used for samples, with consensus chimera removal (chimera_method). The minimum fold change of parent abundance over the chimera for it to be identified (min_fold_parent_over_abundance) was set to 1. The analysis utilized 1 thread (n_threads) and learned error rates from 1,000,000 reads (n_reads_learn), with hashed feature IDs enabled (hashed_feature_ids set to true). Next, the ASV feature table was passed through the feature-classifier plugin [30], which was implemented using a naive Bayes machine-learning classifier, pretrained to discern taxonomy mapped to the latest version of the rRNA database Greengenes2 (2022.10 from 515F/806R region of sequences) [31]. Based on an assessment of alpha (α) rarefaction, a threshold of 10,000 sequences/sample was established. A phylogenic tree was then constructed using the fragment-insertion plugin with Greengenes2 at a *P* sampling depth of the rarefaction threshold to impute high-quality reads and normalize for uneven sequencing depth between samples [32]. α Diversity (intracommunity diversity) was measured using observed ASVs and Faith’s phylogenetic diversity (PD) index. Beta (β) diversity (intercommunity diversity) was measured using weighted and unweighted unique fraction (UniFrac) distances. The estimated functional potential of the overall bacterial community was surveyed via the Phylogenetic Investigation of Communities by Reconstruction of Unobserved States 2 algorithms (v2.4.2) [33]. Pathway abundances were inferred based on structured pathway mappings of Enzyme Commission gene families to the MetaCyc database [34]. Prior to differential abundance analysis at the genus level, microbiota feature data was normalized by sequencing depth, and low abundant taxa were removed (i.e., taxa not present in ≥10% of samples).

### Sample size

Using existing data for grain consumption effects on the primary outcome of LDL cholesterol [35], power calculations (power = 0.90, significance level *P* < 0.05) suggested that a sample size of 37 participants would be sufficient to detect a 7.8% (9.82 mg/dL) difference in LDL cholesterol between treatments assuming a 10% within-person SD. In previous GM studies, including 24–31 participants has been sufficient to see significant treatment differences in community structure [[22], [23], [24]]. Therefore, for 37 people to finish all treatments, after accounting for a 20% drop-out rate, the target recruitment was a total of 45 participants. Because of the unforeseen challenges with recruitment and retention (e.g., participant relocation, COVID-19, etc.), there was a need to overrecruit beyond 45 participants to retain 36 participants who endured the entire 16-wk study timeline. Based on an *ad hoc* power analysis for a linear-mixed model, which assumed no order effect for the aforementioned primary analysis, the trial had 80% power to detect a –2.5 mean difference in cholesterol values, assuming an SD of 5.4 at a 5% significance level with 36 participants.

### Statistical analyses

Participant characteristics are presented as means ± SDs or as percentages and counts where appropriate. The normality of the data was assessed via QQ plots and Shapiro-Wilk tests. Depending on normality, comparisons were made using Student’s t-tests or Wilcoxon rank-sum tests. Spearman’s correlation tests were performed for all association analyses. Nutritional data across groups was assessed by 1-way analysis of variance (ANOVA) tests. Outliers were identified based on *z*-score calculations, defined as values exceeding ±3 SDs from the mean. To maintain the integrity of the longitudinal crossover study data, outliers were replaced with the preceding nonoutlier value for the same participant, ensuring temporal sequence preservation while minimizing distortion from extreme values.

Mixed-effects modeling was used for complete case analysis using the ‘nlme’ package (v3.1.162). The model included fixed effects for treatment, time, period, sequence, and their interactions, aiming to assess variability in outcomes. Random-effects accounted for within-subject correlation across study periods. The significance of fixed effects was tested using ANOVA, focusing on main effects and 2- and 3-way interactions. Post hoc pairwise comparisons for significant interactions were conducted using the Tukey method (‘emmeans’ package, v1.8.8). Model covariance structures were optimized based on the Akaike information criterion and Bayesian information criterion. Residual normality was checked using the Shapiro-Wilk test, and homoscedasticity was assessed via the Breusch-Pagan test (‘lmtest’ package, v0.9.40). A 5-fold cross-validation approach was implemented using ‘caret’ (v6.0.94). Grouped K-fold cross-validation was used, keeping all observations from the same participant in the same fold. The root mean squared error (RMSE) was calculated for each fold, and an mean RMSE provided a measure of the model’s predictive accuracy.

Differences in α diversity and centered log-ratio transformed genus abundance were compared using linear-mixed models, consistent with lipid outcome modeling. β Diversity metrics were assessed via permutational multivariate ANOVA (PERMANOVA) (‘vegan’ package, v2.6.4), incorporating the same terms as in the linear-mixed models. Homogeneity in multivariate dispersion was evaluated using the permutation test for homogeneity in multivariate dispersion. Intraindividual distances for each treatment arm were assessed with Kruskal-Wallis tests. Differences between weighted and unweighted UniFrac metrics were compared using Wilcoxon rank-sum tests.

Changes in Bristol stool and symptom scores were calculated for each participant across treatment periods. Differences between treatment groups were assessed using Kruskal-Wallis tests, with Dunn’s test for post hoc pairwise comparisons where relevant. Additionally, participant satisfaction scores with the 2 food products (pita bread and muffins) consumed during each 4-wk intervention were aggregated for each treatment arm. Scores across the treatment arms and satisfaction category were analyzed using Kruskal-Wallis tests, followed by Dunn’s test for post hoc analysis where appropriate.

Analysis was performed in a blinded fashion. A 2-sided *P* value <0.05 was considered significant, except for microbial taxa abundances, where Benjamini-Hochberg correction was applied for *P* value adjustment (*P*_adj_ < 0.10). All analyses were conducted using R (v4.3.1).

## Results

### Participant characteristics

Of the 131 potential participants who completed the initial blood lipid screening, 54 were randomly assigned to the intervention arms according to random sequence and began consuming study foods. Eighteen participants were lost to follow up, with reasons including decreased interest in continuing the study (*n* = 3), unspecified reasons (*n* = 3), COVID-19 (*n* = 3), relocation out of state (*n* = 2), change in health status (*n* = 2), adverse reaction or discomfort following food consumption (*n* = 2), personal reasons (*n* = 1), antibiotic use (*n* = 1), and became pregnant (*n* = 1). A total of 36 participants completed all 3 treatment arms (Supplemental Figure 1) between March 2018 and August 2023. Table 1 presents baseline demographic, anthropometric, and metabolic characteristics. Of the participants that provided results for the full analysis, the majority were female (∼58%) and White (∼64%). Age ranged from 18 to 67 y old, and BMI (in kg/m^2^) ranged from 18.9 to 40.4. Comparing characteristics by sex, males had a significantly greater body weight (mean difference: +19.8 kg) and height (+12.6 cm) and lower HDL cholesterol (–13.7 mg/dL) in comparison to females (*P* < 0.001). The mean plasma concentrations for baseline LDL cholesterol and TC were both clinically elevated (>100 mg/dL and >200 mg/dL, respectively).

**TABLE 1:** Baseline demographic, anthropometric, and metabolic characteristics of participants.^1^

Characteristics	Total (*n* = 36)	Females (*n* = 21)	Males (*n* = 15)
Age at baseline, y	39.8 ± 13.6	40.3 ± 14.1	40.1 ± 13.4
Race/ethnicity, % (*n*)			
White	63.9 (23)	71.4 (15)	53.3 (8)
Asian	13.9 (5)	9.5 (2)	20.0 (3)
Black	0.0 (0)	0.0 (0)	0.0 (0)
Hispanic	16.7 (6)	14.3 (3)	20.0 (3)
Other	5.6 (2)	4.8 (1)	6.7 (1)
Weight, kg	82.3 ± 18.5	73.6 ± 15.6	93.5 ± 15.6^2^
Height, cm	169.7 ± 8.3	164.6 ± 4.4	177.1 ± 6.5^2^
BMI, kg/m^2^	28.4 ± 5.4	27.2 ± 5.5	29.9 ± 4.9
Total cholesterol, mg/dL	232.0 ± 34.4	231.7 ± 37.2	232.4 ± 31.3
LDL cholesterol, mg/dL	150.7 ± 27.1	147.1 ± 29.0	155.7 ± 24.2
HDL cholesterol, mg/dL	54.3 ± 13.3	60.0 ± 13.6	46.4 ± 7.5^2^
Triglycerides, mg/dL	134.9 ± 52.5	123.1 ± 42.4	151.5 ± 61.7

Abbreviations: BMI, body mass index; HDL cholesterol, high-density lipoprotein cholesterol; LDL cholesterol, low-density lipoprotein cholesterol; SD, standard deviation.

1 Values are displayed as means ± SDs unless stated otherwise.

2 Significant difference between females and males as assessed by student t-test or Wilcoxon rank-sum test depending on normality, *P* < 0.001.

### Treatments

For the 3 treatment arms, compliance was high across the 3 treatment groups (WCM, 96.6 ± 7.9%; RCM + B, 96.7 ± 6.3%; RCM, 95.6 ± 7.2%) and not significantly different (Kruskal-Wallis test, *P* = 0.252). In addition, nutrient intake prior to each intervention was not significantly different across treatment arms (ANOVA tests, *P* ≥ 0.408; Supplemental Table 3). Weight did not significantly change over time between treatments (*P* = 0.985).

### Cardiometabolic markers

**FIGURE 1:**
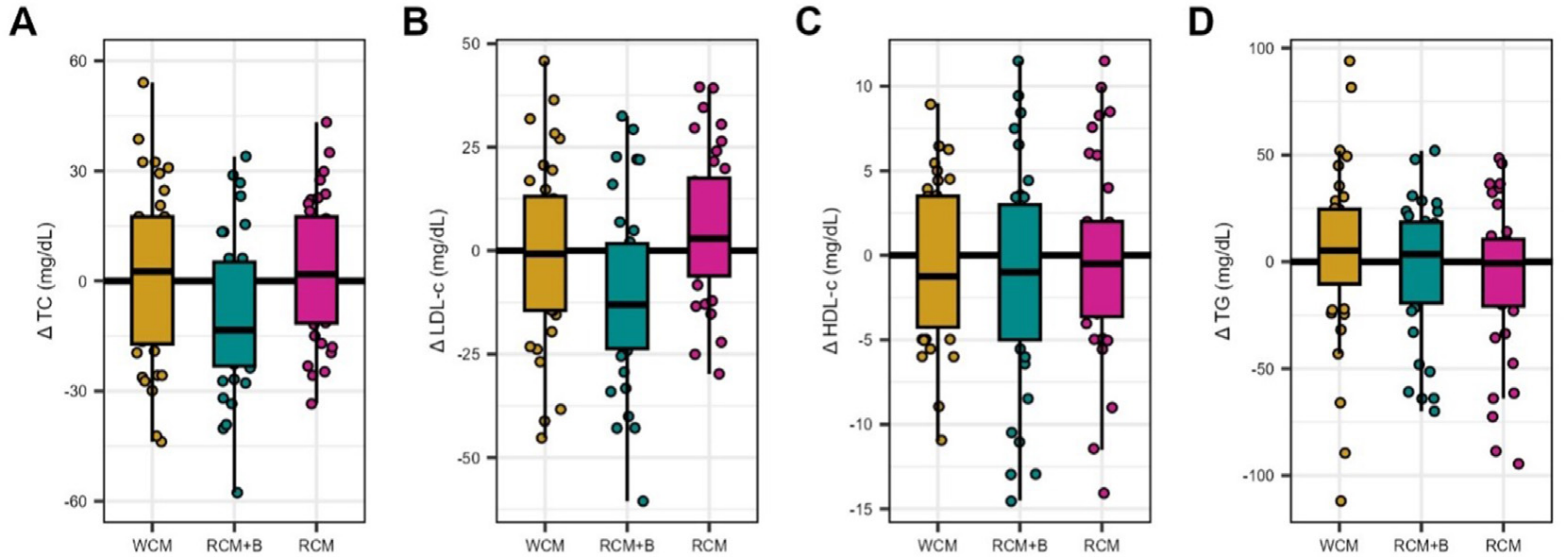
Delta change (time) in lipid metrics by treatment. Delta for (A) total cholesterol (TC), (B) LDL cholesterol, (C) HDL cholesterol, and (D) triglycerides (TG) in mg/dL by treatment group. Boxplots show median, IQR, and outliers for delta changes. Jittered individual points provide a view of data distribution within each treatment group, highlighting variations in treatment response. HDL cholesterol, high-density lipoprotein cholesterol; IQR, interquartile range; LDL cholesterol, low-density lipoprotein cholesterol; RCM: refined corn meal; RCM + B: ∼70:30 blend of RCM and corn bran; WCM: whole-grain corn meal.

In our mixed-effects model analysis assessing the impacts of treatment, time, period, and their interactions on various lipid profiles, several key findings emerged (Figure 1A–D; Supplemental Table 4). For TC and LDL cholesterol, no significant main effects of treatment, time, or period were observed (*F*_(1, 162)_ ≤ 0.923, *P* ≥ 0.361). However, a significant interaction between treatment and time in the LDL cholesterol analysis was detected (*F*_(2, 162)_ = 4.346, *P* = 0.015) as well as a trend toward significance for TC (*F*_(2, 162)_ = 2.676, *P* = 0.072). Pairwise comparisons to further understand this interaction revealed that RCM + B elicited a significant decrease in LDL cholesterol concentrations over time (estimated difference = –10.4 ± 3.6 mg/dL, *P* = 0.005; Figure 1B) with reductions >5% in ∼70% of participants (–22.5 ± 3.0 mg/dL; *n*, 24/36). This was not the case for WCM (estimated difference = –0.6 ± 3.6 mg/dL, *P* = 0.857) or RCM (estimated difference = +4.5 ± 3.6 mg/dL, *P* = 0.209) which resulted in no change. Exploring TC, pairwise comparisons showed a similar pattern as LDL cholesterol (WCM: +1.2 ± 3.8 mg/dL; RCM + B: –9.2 ± 3.9 mg/dL; RCM: +2.5 ± 3.8 mg/dL) with reductions occurring in ∼60% of participants (–22.6 ± 2.7 mg/dL; *n*, 22/36). These pairwise comparisons highlight that the response to RCM + B over time was notably different compared to the other treatments. No other significant interaction effects were observed for TC or LDL cholesterol (*F*_(1, 162)_ ≤ 0.836, *P* ≥ 0.504), though the sequence of treatments exhibited a significant effect on both TC and LDL cholesterol concentrations (*F*_(5, 30)_ ≥ 3.313, *P* ≤ 0.017).

To confirm that the sequence effects were not a result of carryover or treatment order, baseline cholesterol concentrations were compared across the 3 treatment periods, and a period × treatment interaction was explored. No differences were observed for TC and LDL cholesterol across all 3 period baseline values (*P* > 0.980), nor were carryover effects observed (treatment × period effects, *P* ≥ 0.580). Further exploration revealed that the sequence differences were likely driven by variability in mean TC (*P* < 0.007) and LDL cholesterol (*P* < 0.015) concentrations at the very beginning of the study (study visit 1). Post hoc Tukey comparisons yielded significant group differences for TC but not for LDL cholesterol when comparing sequence assignments.

In the case of HDL cholesterol, a different pattern was observed (Figure 1C). The analysis revealed a significant main effect of treatment (*F*_(2, 162)_ = 3.803, *P* = 0.0243) but no notable effects for time, period, sequence, or interactions (*F*_(1, 162)_ ≤ 1.213, *P* ≥ 0.307). To further explore the significant main effect of treatment, pairwise comparisons were conducted (mean change for WCM: –0.7 ± 4.8 mg/dL; RCM + B: –0.9 ± 6.5 mg/dL; RCM: –0.3 ± 5.6 mg/dL), indicating a significant difference between WCM and RCM + B (estimate = 1.7 ± 0.7, *P* = 0.0329) in HDL cholesterol concentrations. However, no significant differences were observed between WCM and RCM (estimate = 0.3 ± 0.7, *P* = 0.9422) or between RCM + B and RCM (estimate = –1.4 ± 0.7, *P* = 0.0736).

Upon assessment of the TG data, 3 outliers were identified (*z*-score >3.0) and were replaced with their respective preceding values. The TG analysis, conducted after adjusting for outliers, indicated no significant effects or interactions [*F*_(1, 162)_ ≤ 1.558, *P* ≥ 0.253; Figure 1D], pointing to a more uniform response across treatments, time periods, and sequences.

Across all lipid profile components, residual diagnostics confirmed the normal distribution of residuals (Shapiro-Wilk W ≤ 0.996, *P* ≥ 0.226), suggesting the appropriateness of the model assumptions. The model’s predictive accuracy, as indicated by the cross-validated RMSE, was found to be moderate for TC (RMSE = 18.52, 144.20–336.81 mg/dL) and LDL cholesterol (RMSE = 17.58, 87.70–226.48 mg/dL), and high for log-transformed HDL cholesterol (RMSE = 0.09, 3.47 –4.51), relative to the respective ranges of these lipid measurements in our dataset. For TG, the predictive accuracy was also moderate when considering the observed range of log-transformed values (RMSE = 0.25, 3.96–5.70 mg/dL).

### GM metrics

In our investigation of GM alterations in response to the dietary interventions, we applied mixed-effects modeling to log-transformed α diversity metrics: Observed ASVs, Pielou’s evenness, and Faith’s PD. This approach paralleled our analysis methodology for lipid biomarkers. However, our findings revealed no significant main or interaction effects for Observed ASVs, Pielou’s evenness, and Faith’s PD (*F*_(1, 162)_ ≤ 1.393, *P* ≥ 0.238; see Supplemental Figure 2A–C). This lack of significant change was consistent across all sequences (*F*_(5, 30)_ ≤ 0.533, *P* ≥ 0.749). The analysis also indicated a normal distribution of residuals (Shapiro-Wilk W ≤ 0.988, *P* ≥ 0.059) alongside high predictive accuracy, underscoring the robustness of our model assumptions. As with TC and LDL cholesterol, no differences were observed for observed ASVs and Faith’s PD across period baseline values (*P* ≥ 0.580), nor were carryover effects observed (treatment × period effects, *P* ≥ 0.239). Similarly, α diversity metrics at the study baseline did not differ by sequence (*P* ≥ 0.749).

To examine the potential influence of gastrointestinal transit time on the GM, we conducted a correlation analysis between α diversity metrics and Bristol stool scores. This analysis revealed no significant correlations [Spearman’s rho (ρ) ≤ –0.041, *P* ≥ 0.389], suggesting that gastrointestinal transit time likely did not significantly impact our microbiota findings. Furthermore, when exploring associations between lipid metrics and α diversity, we observed distinct correlations. Both observed ASVs and Faith’s PD exhibited a positive association with LDL cholesterol concentrations (Spearman’s ρ ≥ 0.178, *P* ≤ 0.012). Conversely, a negative association was noted with TG concentrations for observed ASVs and Faith’s PD (Spearman’s ρ ≥ –0.143, *P* ≤ 0.036). Pielou’s evenness was not significantly associated with LDL cholesterol (Spearman’s ρ = 0.101, *P* = 0.139) or TG concentrations (Spearman’s ρ = –0.123, *P* = 0.071).

In alignment with the trends observed in α diversity, our exploration of β diversity metrics also did not reveal significant effects of treatment or their interactions. Using nested PERMANOVA modeling, we found negligible explained variance for both treatment (*R*^2^ ≤ 0.004, *P* ≥ 0.702) and interaction effects (*R*^2^ ≤ 0.015, *P* ≥ 0.216). Our analysis included both unweighted UniFrac, which accounts for the presence or absence of nondominant microbes, and weighted UniFrac, which considers abundance in addition to presence. Interestingly, treatment sequence (*R*^2^ ≥ 0.059, *P* ≤ 0.005) and participant factors (*R*^2^ ≥ 0.397, *P* ≤ 0.001) emerged as significant, the latter indicating that individual variability was a primary driver of differences in community composition. Further, we computed intra-participant distances between sequential stool collections across different diet treatments. Our findings demonstrated no notable differences between diet treatments in both unweighted and weighted UniFrac intra-participant distances (Kruskal-Wallis tests, *P* ≥ 0.849; refer to Supplemental Figure 2D and E). However, a significant observation was that overall unweighted UniFrac values were considerably higher than weighted UniFrac values (Wilcoxon rank-sum test, *P* < 0.001), suggesting that less abundant microbial species exhibited greater variability within individuals during the study. Additionally, β dispersion analysis for both metrics indicated no significant differences (*P* ≥ 0.673), increasing our confidence that the compositional differences were not an artifact of variance in treatment dispersion.

**FIGURE 2:**
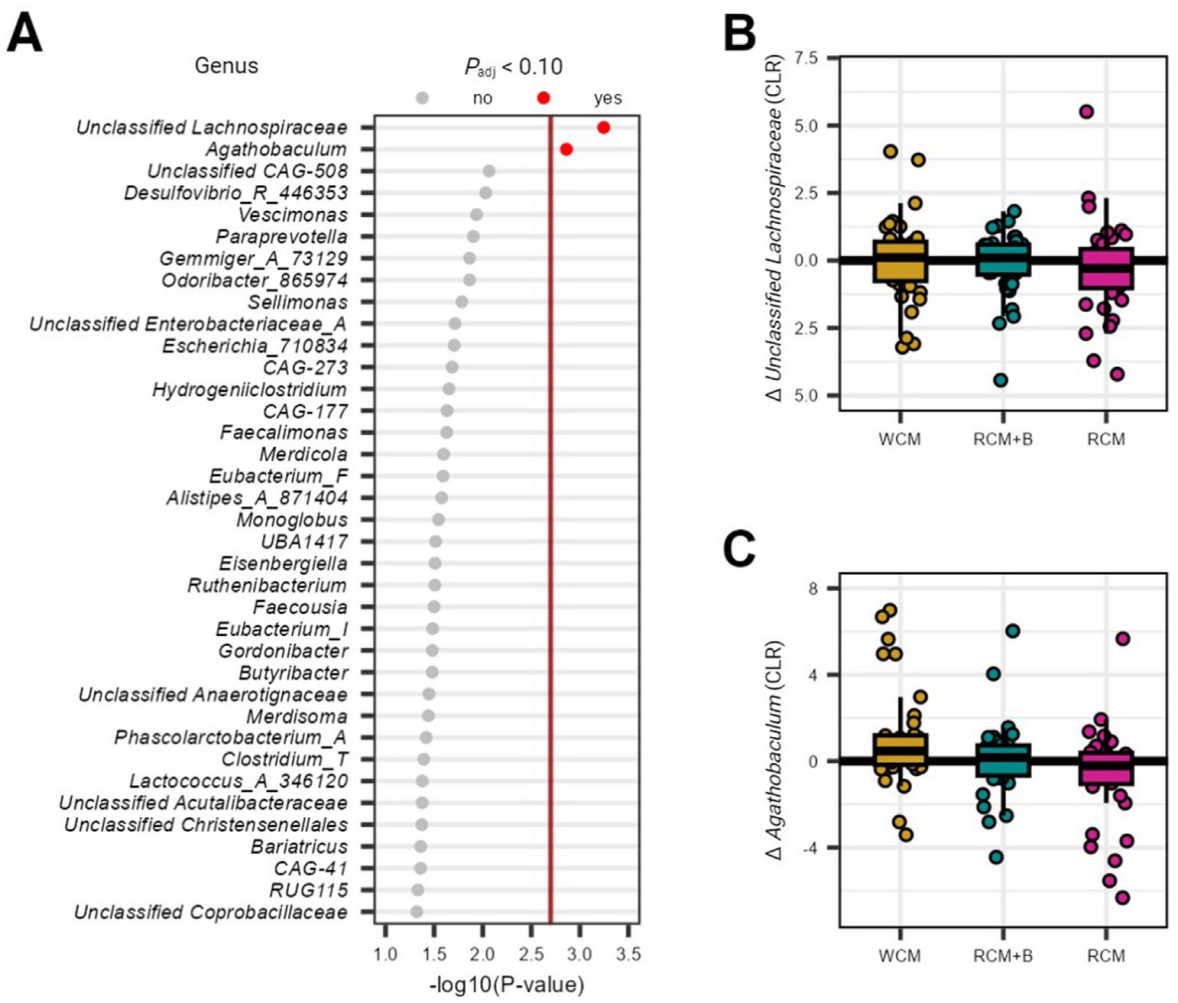
Comparative analysis of genus-level changes in centered log-ratio (CLR) transformed relative abundance across 3 treatment groups. (A) Genus features with a *P* value < 0.05 as determined by mixed model analysis. The red horizontal line marks the threshold for statistical significance, set according to the Benjamini-Hochberg *P* value correction method. Delta for (B) unclassified Lachnospiraceae and (C) Agathobaculum in CLR by treatment group. Boxplots show median, IQR, and outliers for delta changes. Jittered individual points provide a view of data distribution within each treatment group, highlighting variations in treatment response. IQR, interquartile range; RCM: refined corn meal; RCM + B: ∼70:30 blend of RCM and corn bran; WCM: whole-grain corn meal.

Differential abundance analysis at the genus level identified 37 significant features when considering treatment and controlling for participant, time, period, and sequence (Figure 2A; Supplemental Table 5). However, post-adjustment for multiple comparisons yielded only 2 significant genera: an unclassified genus from the *Lachnospiraceae* family (*P*_adj_ = 0.088; Figure 2B) and *Agathobaculum* (*P*_adj_ = 0.096; Figure 2C). The pairwise comparison revealed a notable increase in *Agathobaculum* abundance in WCM compared to RCM (estimated increase = 1.64 ± 0.50 centered log-ratio abundance, *P*_adj_ = 0.025). All other comparisons between treatments did not reach significance (*P*_adj_ ≥ 0.489). Additionally, Spearman correlation analyses to explore associations between these significant taxa and reductions in LDL cholesterol did not demonstrate any significant relationships (*P*_adj_ ≥ 0.106). Finally, assessing estimated functional pathways via Phylogenetic Investigation of Communities by Reconstruction of the Unobserved States 2 assessment did not yield any significant findings (*P*_adj_ ≥ 0.119).

In light of the pronounced interindividual variability observed in GM responses, as evidenced by β diversity analyses, we undertook an exploratory investigation into microbiota responders. We categorized participants based on whether their LDL cholesterol concentrations decreased by >5% following the RCM + B intervention (*n* = 21) – a threshold chosen to mitigate the impact of normal daily variances [36] – compared with those who did not exhibit such reductions (*n* = 15). Preliminary comparisons of diversity and composition, segmented by responder status before RCM + B treatment, showed no significant differences in baseline metrics, including observed ASVs and Faith’s PD (*P* ≥ 0.428), weighted and unweighted UniFrac distances (PERMANOVA, *P* ≥ 0.515), and genus-level taxa (*P*_adj_ ≥ 0.222). Subsequent analyses tracking changes over time across these parameters similarly indicated no significant differences for observed ASVs and Faith’s PD (*P* ≥ 0.706), weighted and unweighted UniFrac intra-individual distances (*P* ≥ 0.076), and genus-level characteristics (*P*_adj_ ≥ 0.516).

### Stool characteristics, gastrointestinal symptoms, and product satisfaction

Changes in Bristol stool scale ratings (post – pre) were not significantly different between treatments (Kruskal-Wallis test, *P* = 0.543; Supplemental Table 6). Similarly, there was no difference between treatments for change in self-reported gastrointestinal symptoms, including increased experience of abdominal pain, bowel movements, bloating, flatulence, fullness, and liquids (Kruskal-Wallis tests, *P* ≥ 0.105; Supplemental Table 7). There was no difference in product satisfaction scores across treatments for the 2 food products (Kruskal-Wallis tests, *P* ≥ 0.71; Supplemental Tables 8 and 9). In addition, both pita and muffin food products were well accepted by participants across all 3 treatment arms with a median rating of “good” [median: 4 (3, 5)] for the categories of appearance, consistency/texture, flavor, and overall satisfaction (5-point scale: 1-unsatisfactory to 5-excellent).

## Discussion

This crossover study was designed to evaluate the effects of consuming 3 corn flour variants (48 g/d: WCM, RCM, and RCM + B) on cardiometabolic outcomes and the GM in adults with elevated LDL cholesterol concentrations (>110 mg/dL). The study revealed a significant reduction in LDL cholesterol concentrations with RCM + B, with an mean decrease of 10 mg/dL (–5.2%). Among RCM + B responders (>5% decrease in LDL cholesterol; 70% of participants experienced reduced LDL cholesterol), substantial reductions in LDL cholesterol concentrations (–22.5 mg/dL or –13.3%) were observed, underscoring individual variability in response to the treatment. Regarding GM, the study observed minimal shifts at the genus level, suggesting that these dietary interventions had a limited impact on gut microbial composition.

Reductions in LDL cholesterol concentrations observed in this study reached clinical significance for LDL cholesterol reduction strategies comparable to other dietary approaches (e.g., almonds and beans) [37,38]. The effectiveness of RCM + B over other flours further suggests the utility of bran-enriched corn products to manage LDL cholesterol, as the observed reduction was comparable to similar studies of grain and fiber consumption [39,40]. To date, the majority of studies investigating cereal bran have focused on oat and rice bran, with limited evidence for corn bran. Of the prior studies, 2 found potential benefits of corn bran in conjunction with other dietary interventions [11,13]. One of the studies found that supplementing a low-fat diet with 20 g/d corn bran for 6 wk resulted in an additional 3.8% reduction in LDL cholesterol beyond the 10.2% reduction afforded by the low-fat diet in hypercholesterolemic males, whereas added wheat bran increased LDL cholesterol by 3.3% [11]. The second study, also in hypercholesterolemic adults, found that 20 g/d of a mixed fiber supplement (guar gum, pectin, soy fiber, pea fiber, and corn bran), compared to placebo, resulted in significant reductions in LDL cholesterol (–12.1% and –11.6% change at 15 and 51wk, respectively) [13]. Collectively, the incorporation of corn bran into food products may be a feasible method of enhancing dietary fiber intake for lowering LDL cholesterol but this approach needs to be explored in conjunction with other lifestyle interventions. In addition, incorporating corn bran into commonly consumed baked goods (like those in our study) and processed food items highlights its practicality and potential as a simple, palatable, and impactful dietary approach to cardiometabolic health management.

Interestingly, the absence of significant LDL cholesterol reductions with whole-grain corn flour contrasts with other whole grains (oats, wheat, rice, rye, barley, and mixed whole grain products) reported cholesterol-lowering effects [6]. Little data are available for the health effects of whole-grain corn, but 1 study evaluating the consumption of a mixture of whole-grain wheat, corn, and rice (∼75%, ∼15%, and ∼10% of food items provided, respectively) products resulted in significant reductions in LDL cholesterol and non-HDL cholesterol when compared to a similar combination of refined grain products [12]. This discrepancy might be attributed to variations in the nutrient composition of different grains, particularly dietary fiber quantity, which in the former study surpassed (>13 g/d) that of the whole-grain corn (∼4 g/d) arm in the current study. Grain mixtures also provide a larger variety of dietary fiber types that, when combined, may provide more robust health benefits.

Contrasting with LDL cholesterol findings, no significant reductions were observed in other lipid parameters (TC, HDL cholesterol, and TG) across treatments. This is in contrast to the study conducted in males with hypercholesterolemia in which corn bran added to a low-fat diet resulted in significant reductions in TC (–5.2%), TG (–13.1%), and very-LDL cholesterol (–13.8%) [11]. Again, the benefits of corn bran were smaller than those afforded by a 2-wk run-in on a low-fat diet (–13.1%). Profiling multiple lipid parameters is crucial for assessing the overall impact of dietary interventions on cardiovascular health, as previous research has reported varying impacts of different whole grain and bran-enriched food products on lipid profiles [40], suggesting that the effects of cereal grain components and fibers may differ by grain type [41].

Regarding GM, our study did not observe significant GM diversity changes in response to corn-based product consumption. However, past research has indicated shifts in GM, such as a study of females and males receiving 25 g/d and 35 g/d of corn bran arabinoxylan, respectively, resulting in significant alterations in the GM community structure [17]. Beyond diversity, prior work has reported increased *bifidobacteria* abundance with corn-based whole-grain cereal consumption [16], whereas consumption of whole-grain wheat, corn, and rice products in combination resulted in no significant differences in taxa abundance [12]. Compliance and fiber dose may have driven these differential effects as high consumers from the mixed product study showed an increased abundance of *Akkermansia* and *Lactobacillus* [12]. Large-scale microbiota surveys, such as results from the American Gut Project, have revealed significant positive associations between whole grain consumption and microbial diversity, as measured by the Healthy Eating Index-2015 scores [42]. However, a review by Koecher et al. [43] (2019) on the impact of various whole grains (wheat, barley, rye, rice, corn, oats, or mixtures) on the GM highlighted inconsistencies across measures of microbial diversity and taxa. These findings underscore the need for future studies to compare the effects of individual grains and mixtures of fibers on GM to better understand the dietary impacts on microbial community structure and function.

The dynamic and complex nature of GM also requires further exploration to fully understand the interplay between fiber doses and microbial community structure. Diets rich in microbiota-accessible carbohydrates can reach the microbial-dense regions of the colon largely intact, where they are metabolized, resulting in the production of beneficial organic acids like butyrate [[44], [45], [46]]. Recent short-term studies with diets rich in microbiota-accessible carbohydrates have reported significant community shifts over time [18] and in comparison to a low-fiber Western diet [44]. However, these interventions involved participants substantially increasing their dietary intake (e.g., ∼40–50 g fiber per day). Although we did not observe significant community shifts (i.e., β diversity) in the present study, the increase in *Agathobaculum* abundance following WCM consumption is notable due to its butyrate-producing capability and subsequent importance for gut health [47]. The relationship between this microbial genera and whole-grain corn consumption is interesting as it conflicts with studies of extracted type-4 resistant starch derived from high-amylose maize starch, which reduced *Agathobaculum butyriciproducens* abundance [48]. This difference likely stems from the unique effects of grain extracts compared with whole components of the kernel, as well as how food matrix (mixed ingredient food products compared with supplements) effect the GM. *Agathobaculum* has been found to increase in adults consuming higher fiber diets. For example, *Agathobaculum butyriciproducens* increased after daily guar gum ingestion (8 g for 18 d) in healthy males [49], as well as in healthy adults consuming a fiber-enriched Mediterranean-type diet [50]. *Agathobaculum* requires further investigation as a potential microbial target for grain interventions and cardiovascular health improvements.

Correlations observed between LDL cholesterol, TG, and GM diversity suggest a complex interplay influenced by dietary interventions. The GM’s role in cholesterol metabolism, impacting LDL cholesterol concentrations through various mechanisms, is a significant area of interest. Notable processes include its involvement in the formation of short-chain fatty acids and the synthesis of bile acids [51]. Additionally, gut microbes contribute by diminishing the absorption of cholesterol in the intestine [52]. Enzymes also play a part in reducing the amount of absorbed cholesterol [52]. More recently, a comprehensive profile of stool metagenomics and metabolomics from 1429 Framingham Heart Study participants revealed species from the *Oscillibacter* genus were associated with decreased fecal and plasma cholesterol concentrations [53]. These microbes appear to harbor cholesterol-metabolizing capabilities, including glycosylation and dehydrogenation. However, these findings are cross-sectional and likely reflect long-term dietary and lifestyle factors. Our study did not find changes in these microbes (or the presence of *Oscillobacter,* specifically) nor estimated functional pathways, likely due to its brief duration, small sample size, and the specific dietary components tested. Many of the current studies linking cholesterol metabolism to GM have not focused on how diet mediates these relationships; therefore, further studies are necessary to comprehensively grasp the best modes of grain product delivery and the clinical implications of this information. The results from the present study reinforce the importance of incorporating bran-enriched corn flour in diets for individuals at higher cardiovascular disease risk, thereby supporting evidence-based nutrition recommendations [54].

Strengths of the study include its crossover design, allowing each participant to act as their own control and ensuring a more robust comparison among treatments. High dietary compliance across treatments, monitored by both calendars and returned food products, adds credibility to our findings. The use of baked goods as a delivery method ensures practicality and applicability to general dietary patterns. On the contrary, limitations, such as the small sample size and observed sequence effect for some outcomes, are acknowledged. Future studies should attempt to balance baseline LDL cholesterol concentrations across the study sequences. Additionally, it is important to note that the subtle microbiota changes could have been due to transient changes observed with initial dietary changes. Future studies should explore GM effects over longer time periods with repeated sampling to confirm these findings.

In conclusion, we showed that RCM + B effectively lowered LDL cholesterol concentrations in individuals with elevated cholesterol concentrations. This reduction is clinically meaningful and supports the use of bran-enriched flours as part of a regular component of dietary intake for promoting cardiovascular health. In relation to the GM, we found minimal effects between treatments and high levels of inter-participant variability, highlighting the complex and personal nature of gastrointestinal microbial populations. Further investigation into the mechanisms influencing cholesterol is warranted, given the variability in effects observed in prior literature among different grains and their impact on GM. Additionally, future studies with larger and more culturally diverse samples would benefit from studying other corn-based staple foods (e.g., tortillas), as well as variations in the genetic profiles of participants. Such broader-focused studies would inform health policy and aid in understanding the underlying mechanisms of corn flour on hyperlipidemia.

## Author contributions

The authors’ responsibilities were as follows – CMW, SV-L: designed research; CMW, SV-L, CPO-S, SLW, MK, BT, BL, MH: conducted research; AEM, CPO-S, SLW, MK, BL, MH: managed data; AEM, CMW: analyzed data; CMW, AEM, BL: wrote the article; CMW, SV-L: had primary responsibility for the final content; and all authors: read and approved the final manuscript.

## Conflict of interest

CMW has previously served as a member of Scientific Advisory Boards for 2 grain organizations/businesses: Ardent Mills, LLC and Wheat Foods Council. CMW is currently serving on the Avocado Nutrition Science Advisory for Hass Avocados and the California Walnut Commission’s Scientific Advisory Board.

## Funding

The Corn Division of the North American Millers' Association generously provided funding for this research through the Arizona State University Foundation. The Corn Division provided all corn meals and milled bran as an in-kind donation. The Corn Division reviewed the proposed study for its funding potential but did not have any oversight over the study design, collection, analysis, and interpretation of data, nor writing and revising the manuscript for publication. Additionally, a portion of the time preparing this manuscript was supported by 2 training grants: National Institute of Diabetes and Digestive and Kidney Diseases of the National Institutes of Health under Award Number T32DK137525 (AEM) and HRSA Maternal and Child Health Bureau Award Number T79MC49101 (BL). The content is solely the responsibility of the authors and does not necessarily represent the official views of the National Institutes of Health or the Maternal and Child Health Bureau.

## Data availability

Study participants did not provide consent to publically share data. Therefore, data described in the manuscript, code book, and analytic code can only be made available upon request pending application and approval of the Corresponding Authors and relevant IRB approval for data sharing and analysis.
