# Supplementary material for: Evaluating the Effects of Corn Flour Product Consumption on Cardiometabolic Outcomes and the Gut Microbiota in Adults with Elevated Cholesterol: A Randomized Crossover
Source: J Nutr. 2024 Jun 14;154(8):2437–47. doi: 10.1016/j.tjnut.2024.06.003 (PMC11923428; doi:10.1016/j.tjnut.2024.06.003)
Supplement: Multimedia component 1 [file mmc1.docx]

**Table of Contents**

**Supplemental Figure S1.** Consolidated Standards of Reporting Trials flow diagram………………………………………………...**S-2**

**Supplemental Figure S2.** Delta change (time) in alpha and beta diversity gut microbiome metrics by treatment**S-3**

**Supplemental Table S1.** Ingredients and recipes for study food products**S-4**

**Supplemental Table S2.** Nutritional data for the food products given for each treatment**S-5**

**Supplemental Table S3.** Daily nutrient intakes prior to the three treatment arms for participants with adequate dietary data **S-6**

**Supplemental Table S4.** Pre and post body weight and cardio-metabolic outcomes for each dietary treatment**S-7**

**Supplemental Table S5.** Pre and post mean relative abundance at the Genus-level for each dietary treatment **S-8**

**Supplemental Table S6.** Self-reported stool consistency, as assessed by the Bristol Stool Chart**S-12**

**Supplemental Table S7.** Gastrointestinal symptom prevalence pre- and post-intervention for the three treatment arms**S-13**

**Supplemental Table S8.** Reported satisfaction of pita food product for the three treatment arms**S-14**

**Supplemental Table S9.** Reported satisfaction of muffin food product for the three treatment arms**S-15**

**Supplemental Material: Figures**

**
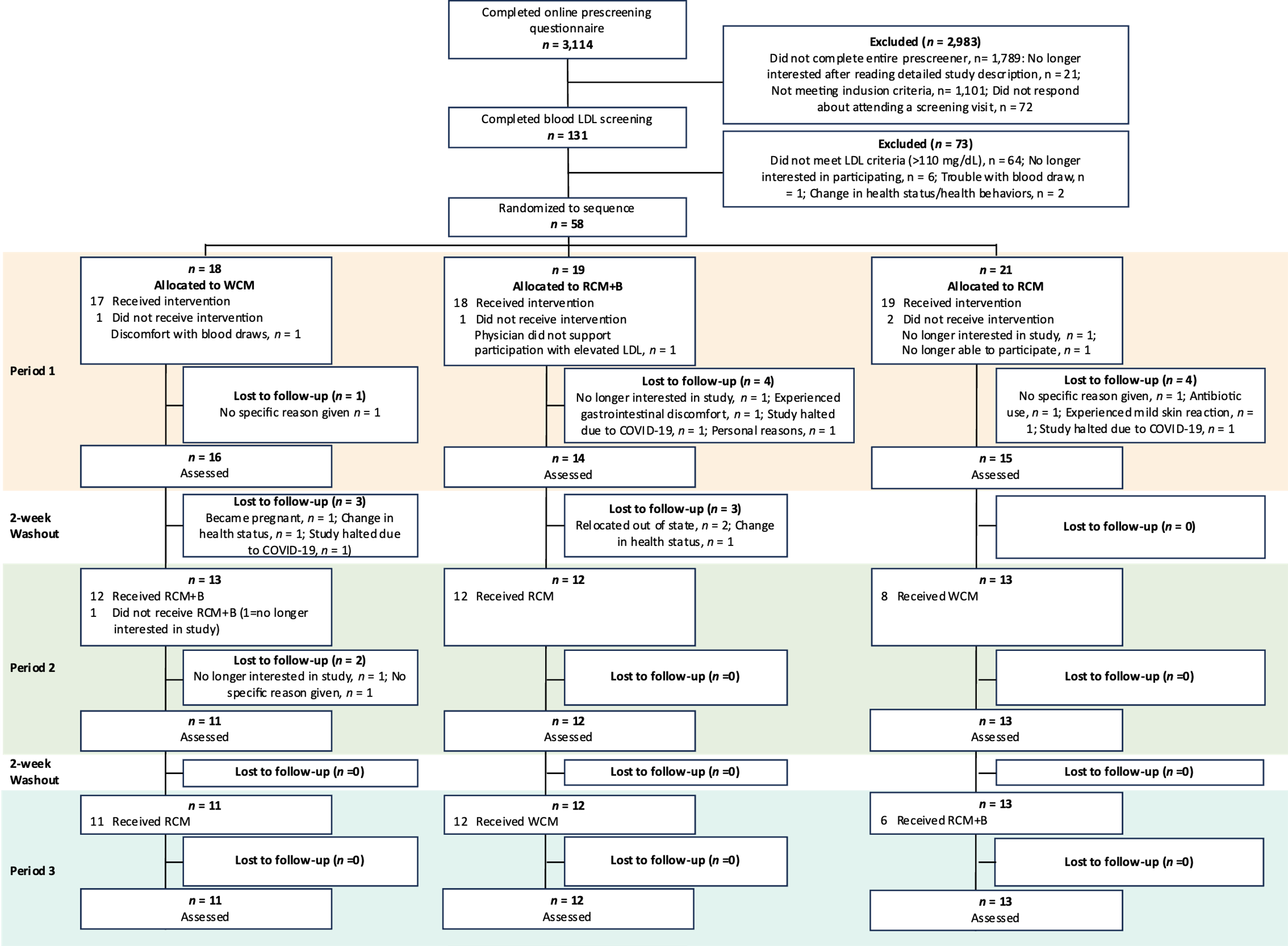
**

**Figure S1.** Consolidated Standards of Reporting Trials flow diagram.

**Supplemental Material: Figures**

**
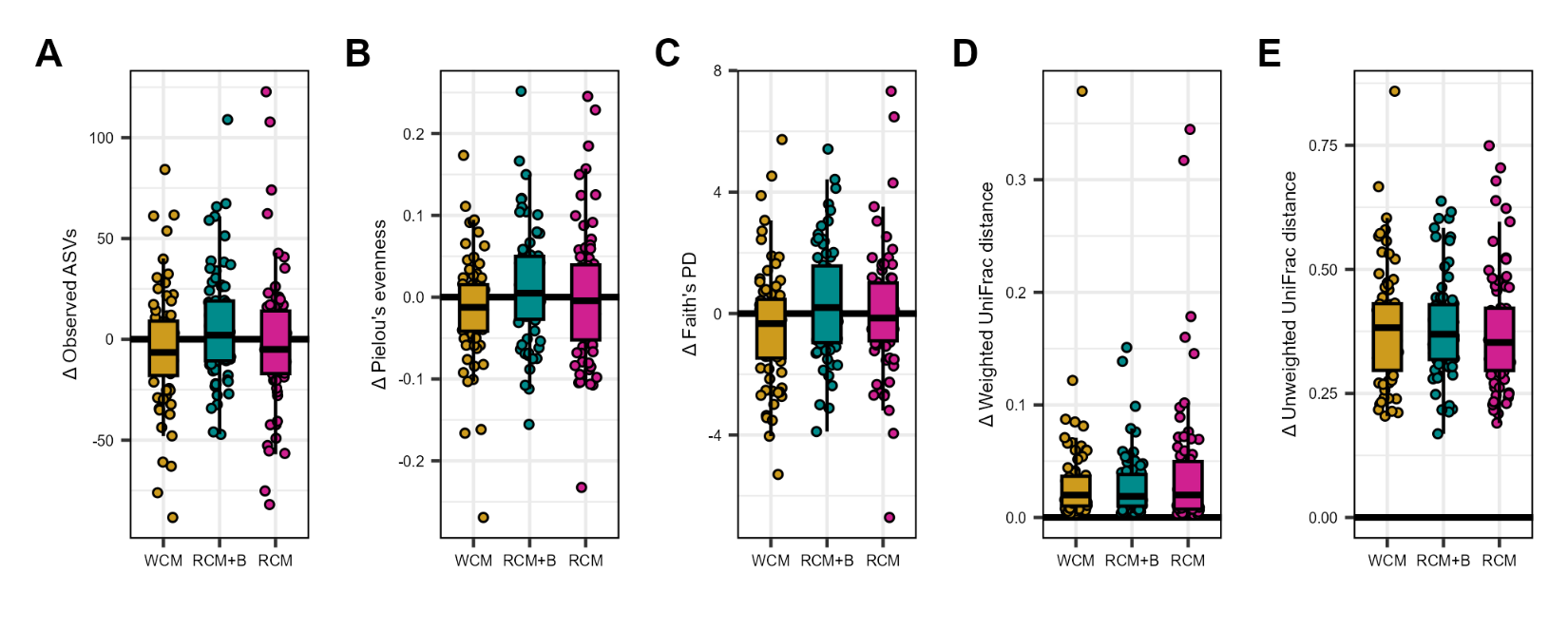
**

**Figure S2.** Delta change (time) in alpha and beta diversity gut microbiome metrics by treatment. Delta for (**A**) observed amplicon sequence variants (ASVs), (**B**) Pielou’s evenness, (**C**) Faith’s phylogenetic diversity (PD), (**D**) weighted UniFrac distance, and (**E**) unweighted UniFrac distance by treatment group. Boxplots show median, interquartile range, and outliers for delta changes. Jittered individual points provide a view of data distribution within each treatment group, highlighting variations in treatment response. WCM: whole grain corn meal; RCM+B: blend of ~70:30 blend of refined corn meal and corn bran by weight; RCM: Refined corn meal.

**Supplemental Material: Tables**

**Table S1.** Ingredient amounts and recipes for individual food products given for each study treatment.

| **Corn Muffin** | |  | **Pita Bread** | |
| --- | --- | --- | --- | --- |
| Ingredient | Grams |  | Ingredient | Grams |
| Water | 29.3 |  | Water | 33.92 |
| Corn flour/meal | 24.06 |  | Corn flour/meal | 24.2 |
| Whole eggs | 18.5 |  | Wheat gluten | 8.08 |
| Sweetex high-ratio icing shortening | 11.4 |  | Crisco shortening | 3.55 |
| Granulated Sugar | 8.3 |  | Granulated sugar | 3.23 |
| Wheat gluten | 6.8 |  | Dry Yeast Gold | 0.65 |
| Honey | 6.2 |  | Salt | 0.58 |
| Baking Powder | 2.2 |  | Baking powder | 0.32 |
| Cargill maltodextrin 01956 | 1.5 |  | Lecithin | 0.32 |
| Non Fat Dry Milk | 1.2 |  | AB Mauri ICS 56 dough enhancer | 0.081 |
| Polartex Instant starch 12640 | 0.8 |  | AB Mauri Softase 4040 enzyme | 0.081 |
| Vanilla extract | 0.6 |  | INSTRUCTIONS:  Put the corn flour, gluten, yeast, and water into mixing bowl, mix, and let rest for 10 minutes. Add the rest of the ingredients and mix to fully develop the gluten. Cut dough into 75 gram pieces and roll into balls. Let rolled balls sit for 15 minutes. Roll each ball out with rolling pin or sheet with sheeter to a thickness of 4 to 5 cm. Put onto pan with parchment paper, dock the dough with fork or docker. Proof for 30 to 60 minutes or until disks double in height. Bake at 375 to 400 F until done, ~ XX min. | |
| Salt | 0.6 |  |  |  |
| Cargill Native starch 03420 | 0.3 |  |  |  |
| AB Mauri Softase 4040 enzyme | 0.1 |  |  |  |
| INSTRUCTIONS:  Mix the shortening, sugar, salt, starches, maltodextrin and dry milk on low just until ingredients are incorporated. Turn mixer to medium speed and cream for 2 minutes. Add the eggs and vanilla to mixer, scrape down the bowl and paddle, and mix for one more minute on low. Scrape down the sides of bowl and mix again for 2 minutes on low. Stir together gluten, corn, enzymes and baking powder in separate bowl and then mix into main bowl ingredients on low. Slowly add in the water over 1 minute while continuing to mix on low. Scrape down the bowl and paddle, and mix for 2 additional minutes on low. Deposit 112 grams of batter into paper-lined cupcake pans and back at 375 F until done, ~25 min. | |  |  |  |

**Supplemental Material: Tables**

**Table S2.** Nutritional data for the food products given for each study treatment.^1^

| **Variables** | **WCM** | | **RCM+B** | | **RCM** | |
| --- | --- | --- | --- | --- | --- | --- |
|  | Pita (serv.) | Muffin (serv.) | Pita (serv.) | Muffin (serv.) | Pita (serv.) | Muffin (serv.) |
| Energy, kcal | 166 | 310 | 156 | 301 | 168 | 312 |
| Carbohydrate, g | 23.3 | 36.8 | 24.2 | 37.9 | 23.9 | 37.4 |
| Dietary fiber, g | 2.0 | 1.8 | 6.8 | 6.6 | 1.2 | 1.0 |
| Soluble, g | 0.4 | 0.2 | 0.4 | 0.3 | 0.3 | 0.2 |
| Insoluble, g | 1.6 | 1.6 | 6.2 | 6.2 | 0.8 | 0.8 |
| Fat, g | 4.9 | 14.4 | 4.4 | 13.9 | 4.5 | 13.9 |
| Saturated, g | 1.1 | 3.6 | 1.0 | 3.5 | 1.0 | 3.5 |
| Protein, g | 8.3 | 9.8 | 8.1 | 9.7 | 8.1 | 9.6 |
| Cholesterol, mg | 0 | 69.0 | 0 | 69.0 | 0 | 69.0 |
| Minerals |  |  |  |  |  |  |
| Sodium, mg | 271.0 | 507.0 | 264.0 | 501.0 | 264.0 | 501.0 |
| Potassium, mg | 84.0 | 124.0 | 41.0 | 82.0 | 49.0 | 90.0 |
| Magnesium, mg | 34.0 | 37.0 | 13.0 | 16.0 | 11.0 | 14.0 |
| Phosphorus, mg | 90.0 | 168.0 | 54.0 | 132.0 | 56.0 | 134.0 |
| Calcium, mg | 33.0 | 166.0 | 35.0 | 168.0 | 32.0 | 165.0 |
| Vitamins |  |  |  |  |  |  |
| Vitamin E, IU | 1.5 | 4.7 | 1.4 | 4.6 | 1.4 | 4.6 |
| Vitamin A, IU | 52.0 | 174.0 | 41.0 | 163.0 | 52.0 | 174.0 |
| Niacin, mg | 1.1 | 0.9 | 1.3 | 1.1 | 1.5 | 1.2 |
| Folate, mcg | 21.0 | 15.0 | 50.0 | 44.0 | 66.0 | 59.0 |

^1^Nutritional composition is based on estimates provided by Nutritional Data System for Research software following recipe analysis with each flour.

**Supplemental Material: Tables**

**Table S3.** Daily nutrient intakes prior to the three treatment arms for participants with adequate dietary data.^1-3^

| **Variables** | **WCM (*n* = 28)** | **RCM+B (*n* = 28)** | **RCM (*n* = 28)** |
| --- | --- | --- | --- |
| Energy, kcal | 1,646 ± 484 | 1,664 ± 577 | 1,763 ± 594 |
| Carbohydrate, g | 175.6 ± 93.1 | 170.6 ± 81.0 | 192.2 ± 91.3 |
| Dietary fiber, g | 15.3 ± 5.7 | 16.5 ± 6.6 | 17.5 ± 6.3 |
| Soluble, g | 4.8 ± 2.1 | 5.1 ± 2.3 | 5.5 ± 2.3 |
| Insoluble, g | 10.4 ± 4.3 | 11.2 ± 5.1 | 11.8 ± 4.8 |
| Fat, g | 70.3 ± 19.9 | 74.8 ± 25.5 | 75.1 ± 21.9 |
| Saturated, g | 20.7 ± 6.8 | 22.4 ± 8.8 | 22.4 ± 7.3 |
| Protein, g | 78.1 ± 31.4 | 78.8 ± 33.3 | 82.4 ± 30.1 |
| Cholesterol, mg | 268.5 ± 194.4 | 253.5 ± 180.7 | 265.1 ± 168.6 |
| Minerals |  |  |  |
| Calcium, mg | 675.9 ± 348.8 | 687.4 ± 344.6 | 735.3 ± 305.2 |
| Copper, mg | 1.1 ± 0.5 | 1.1 ± 0.6 | 1.04 ± 0.4 |
| Iron, mg | 12.6 ± 6.9 | 11.1 ± 4.8 | 13.3 ± 7.7 |
| Magnesium, mg | 246.7 ± 81.9 | 256.9 ± 115.4 | 259.2 ± 93.0 |
| Phosphorus, mg | 1,035.9 ± 295.9 | 1,055.3 ± 386.7 | 1,114.3 ± 366.0 |
| Potassium, mg | 1,935.1 ± 584.5 | 2,051.2 ± 808.5 | 2,075.7 ± 670.1 |
| Selenium, mcg | 115.7 ± 68.2 | 122.3 ± 48.9 | 110.9 ± 48.7 |
| Sodium, mg | 2,842.1 ± 1,479.3 | 2,888.0 ± 1,430.7 | 3,038.7 ± 1,463.1 |
| Zinc, mg | 9.7 ± 3.2 | 9.6 ± 3.4 | 10.1 ± 3.9 |
| Vitamins |  |  |  |
| B1 (Thiamin), mg | 1.6 ± 1.1 | 1.6 ± 1.1 | 2.0 ± 1.6 |
| B2 (Riboflavin), mg | 1.9 ± 1.2 | 1.9 ± 1.2 | 1.9 ± 1.2 |
| B3 (Niacin), mg | 22.3 ± 9.7 | 22.4 ± 11.2 | 25.0 ± 10.5 |
| B5 (Pantothenic acid), mg | 5.2 ± 2.2 | 5.1 ± 2.1 | 5.1 ± 1.8 |
| B6 (Pyridoxine), mg | 1.9 ± 1.8 | 1.9 ± 1.6 | 1.9 ± 1.3 |
| B12 (Cobalamin), mg | 3.9 ± 3.2 | 3.3 ± 2.2 | 3.7 ± 2.2 |
| Folate, mcg | 333.6 ± 149.3 | 322.7 ± 147.6 | 381.1 ± 194.5 |
| Vitamin A, IU | 4,498 ± 3,461 | 3,804.9 ± 2,644.1 | 4,043.7 ± 2,753.9 |
| Vitamin C, mg | 45.9 ± 32.1 | 48.9 ± 40.5 | 46.8 ± 24.6 |
| Vitamin D, mcg | 3.4 ± 3.0 | 3.5 ± 3.7 | 3.4 ± 3.1 |
| Vitamin E, mg | 8.0 ± 4.9 | 9.1 ± 6.1 | 8.2 ± 4.6 |
| Vitamin K, mcg | 95.9 ± 75.2 | 105.8 ± 100.4 | 111.1 ± 93.4 |

^1^Habitual dietary intake is based on estimates provided by Nutritional Data System for Research software after dietary records were entered into the nutrition analysis software.

^2^Values are displayed as means ± SDs, unless stated otherwise.

^3^Data from 8 participants was not complete and thus, not included.

**Supplemental Material: Tables**

**Table S4.** Pre and post body weight and cardio-metabolic outcomes for each dietary treatment.

| **Variables** | **WCM (*n* = 36)** | | **RCM+B (*n* = 36)** | | **RCM (*n* = 36)** | |
| --- | --- | --- | --- | --- | --- | --- |
|  | Pre | Post | Pre | Post | Pre | Post |
| Weight, kg | 81.9 ± 18.1 | 82.1 ± 17.7 | 82.8 ± 16.9 | 82.1 ± 17.4 | 81.9 ± 18.2 | 82.0 ± 17.5 |
| TC, mg/dL | 231.0 ± 37.5 | 232.4 ± 41.3 | 235.5 ± 35.3 | 227.2 ± 28.2 | 231.9 ± 32.9 | 234.5 ± 31.7 |
| LDL-c, mg/dL | 148.6 ± 30.7 | 148.1 ± 31.1 | 154.3 ± 29.1 | 144.8 ± 25.0 | 149.7 ± 25.9 | 154.2 ± 25.7 |
| HDL, mg/dL | 54.7 ± 13.3 | 54.0 ± 13.4 | 53.1 ± 14.5 | 52.0 ± 11.4 | 54.2 ± 13.7 | 53.8 ± 12.3 |
| TG, mg/dL | 138.7 ± 61.9 | 151.4 ± 81.1 | 140.5 ± 55.6 | 151.8 ± 66.6 | 140.4 ± 58.8 | 132.3 ± 50.8 |

**Supplemental Material: Tables**

**Table S5.** Pre and post mean relative abundance at the Genus-level for each dietary treatment.^1^

| **Genus** | **WCM (*n* = 36)** | | **RCM+B (*n* = 36)** | | **RCM (*n* = 36)** | |
| --- | --- | --- | --- | --- | --- | --- |
|  | Pre | Post | Pre | Post | Pre | Post |
| *Acetatifactor* | 0.00185 | 0.00163 | 0.00197 | 0.00220 | 0.00255 | 0.00148 |
| *Acutalibacter* | 0.00054 | 0.00050 | 0.00092 | 0.00049 | 0.00110 | 0.00083 |
| *Adlercreutzia_404257* | 0.00049 | 0.00043 | 0.00079 | 0.00045 | 0.00063 | 0.00060 |
| *Agathobacter_164117* | 0.02660 | 0.02518 | 0.02994 | 0.03152 | 0.03632 | 0.03121 |
| *Agathobaculum* | 0.00293 | 0.00492 | 0.00515 | 0.00570 | 0.00455 | 0.00387 |
| *Akkermansia* | 0.00830 | 0.01100 | 0.01705 | 0.01154 | 0.01904 | 0.01833 |
| *Alistipes_A_871400* | 0.01063 | 0.00647 | 0.01134 | 0.01294 | 0.01221 | 0.00805 |
| *Alistipes_A_871404* | 0.00046 | 0.00039 | 0.00083 | 0.00066 | 0.00117 | 0.00063 |
| *Anaerobutyricum* | 0.02922 | 0.02829 | 0.03085 | 0.02883 | 0.03293 | 0.03311 |
| *Anaeromassilibacillus* | 0.00027 | 0.00020 | 0.00038 | 0.00024 | 0.00040 | 0.00030 |
| *Anaerostipes* | 0.03158 | 0.03338 | 0.03499 | 0.02962 | 0.03494 | 0.03343 |
| *Anaerotignum_189125* | 0.00131 | 0.00085 | 0.00091 | 0.00078 | 0.00061 | 0.00068 |
| *Angelakisella* | 0.00027 | 0.00023 | 0.00071 | 0.00025 | 0.00097 | 0.00084 |
| *Bacteroides_H* | 0.05882 | 0.03495 | 0.04403 | 0.04968 | 0.03792 | 0.03081 |
| *Bariatricus* | 0.00825 | 0.00802 | 0.00760 | 0.00844 | 0.00679 | 0.00752 |
| *Bifidobacterium_388775* | 0.05161 | 0.05630 | 0.06344 | 0.05870 | 0.04687 | 0.06764 |
| *Bilophila* | 0.00087 | 0.00098 | 0.00105 | 0.00144 | 0.00107 | 0.00055 |
| *Blautia_A_141780* | 0.00672 | 0.00754 | 0.00509 | 0.00654 | 0.00713 | 0.00721 |
| *Blautia_A_141781* | 0.17757 | 0.17771 | 0.18593 | 0.18894 | 0.19098 | 0.20225 |
| *Butyribacter* | 0.00097 | 0.00042 | 0.00118 | 0.00072 | 0.00042 | 0.00060 |
| *BX12* | 0.00038 | 0.00028 | 0.00022 | 0.00030 | 0.00028 | 0.00031 |
| *CAG-127* | 0.00078 | 0.00021 | 0.00034 | 0.00035 | 0.00080 | 0.00027 |
| *CAG-1427* | 0.00254 | 0.00268 | 0.00242 | 0.00174 | 0.00249 | 0.00191 |
| *CAG-177* | 0.00066 | 0.00078 | 0.00097 | 0.00065 | 0.00095 | 0.00105 |
| *CAG-217* | 0.00154 | 0.00066 | 0.00041 | 0.00029 | 0.00024 | 0.00055 |
| *CAG-238* | 9.31e-05 | 9.20e-05 | 0.00016 | 9.83e-05 | 0.00012 | 0.00011 |
| *CAG-269* | 0.00132 | 0.00241 | 0.00262 | 0.00085 | 0.00115 | 0.00115 |
| *CAG-273* | 0.00028 | 0.00045 | 0.00033 | 0.00019 | 0.00038 | 0.00027 |
| *CAG-274* | 0.00013 | 0.00024 | 0.00058 | 0.00027 | 0.00030 | 0.00079 |
| *CAG-302* | 0.00043 | 0.00124 | 0.00158 | 0.00068 | 0.00179 | 0.00191 |
| *CAG-317_146760* | 0.00068 | 0.00076 | 0.00065 | 0.00103 | 0.00051 | 0.00076 |
| *CAG-41* | 0.00463 | 0.00656 | 0.00509 | 0.00511 | 0.00524 | 0.00500 |
| *CAG-95* | 7.24e-05 | 0.00014 | 0.00024 | 3.02e-05 | 3.68e-05 | 4.23e-05 |
| *Catenibacillus* | 0.00016 | 0.00029 | 7.93e-05 | 0.00014 | 0.00014 | 0.00019 |
| *Clostridium_A* | 0.00100 | 0.00199 | 0.00213 | 0.00193 | 0.00227 | 0.00187 |
| *Clostridium_AP* | 0.00113 | 0.00051 | 0.00081 | 0.00070 | 0.00082 | 0.00090 |
| *Clostridium_AQ* | 0.00040 | 0.00022 | 0.00066 | 0.00059 | 0.00037 | 0.00065 |
| *Clostridium_Q_134516* | 0.00024 | 0.00014 | 0.00014 | 0.00023 | 9.24e-05 | 5.35e-05 |
| *Clostridium_Q_135822* | 0.00170 | 0.00198 | 0.00141 | 0.00225 | 0.00111 | 0.00109 |
| *Clostridium_T* | 0.00886 | 0.00869 | 0.00668 | 0.00700 | 0.01291 | 0.00807 |
| *COE1* | 0.00017 | 0.00030 | 0.00041 | 0.00027 | 0.00034 | 0.00051 |
| *Collinsella* | 0.01219 | 0.01099 | 0.00931 | 0.00912 | 0.00914 | 0.01185 |
| *Coprobacillus* | 8.67e-05 | 0.00023 | 0.00025 | 0.00012 | 9.80e-05 | 2.47e-05 |
| *Coprococcus_A_121497* | 0.00790 | 0.00426 | 0.00599 | 0.00579 | 0.00563 | 0.00581 |
| *Coprococcus_A_187866* | 0.00405 | 0.00350 | 0.00337 | 0.00408 | 0.00395 | 0.00322 |
| *Copromonas* | 0.00171 | 0.00108 | 0.00168 | 0.00183 | 0.00127 | 0.00100 |
| *Copromorpha* | 0.00112 | 0.00148 | 0.00150 | 0.00153 | 0.00133 | 0.00124 |
| *Desulfovibrio_R_446353* | 0.00091 | 0.00045 | 0.00078 | 0.00099 | 0.00052 | 0.00059 |
| *Dialister* | 0.00248 | 0.01266 | 0.00726 | 0.00711 | 0.00631 | 0.00635 |
| *Dorea_A* | 0.02806 | 0.03099 | 0.03204 | 0.02711 | 0.02715 | 0.04849 |
| *Dysosmobacter* | 0.00181 | 0.00151 | 0.00197 | 0.00170 | 0.00183 | 0.00129 |
| *Eggerthella* | 0.00028 | 0.00028 | 0.00036 | 0.00037 | 0.00022 | 0.00031 |
| *Eisenbergiella* | 0.00214 | 0.00633 | 0.00275 | 0.00239 | 0.00281 | 0.00123 |
| *Ellagibacter* | 0.00018 | 0.00020 | 0.00022 | 0.00029 | 0.00029 | 0.00023 |
| *Enterenecus* | 0.00032 | 0.00036 | 0.00017 | 0.00028 | 0.00036 | 0.00030 |
| *Enterocloster* | 0.00577 | 0.00448 | 0.00341 | 0.00482 | 0.00222 | 0.00300 |
| *ER4* | 0.00197 | 0.00205 | 0.00257 | 0.00302 | 0.00315 | 0.00275 |
| *Erysipelatoclostridium* | 0.00148 | 0.00207 | 0.00222 | 0.00191 | 0.00150 | 0.00347 |
| *Escherichia_710834* | 0.01265 | 0.00272 | 0.00495 | 0.00341 | 0.00463 | 0.01318 |
| *Eubacterium_F* | 0.00160 | 0.00133 | 0.00138 | 0.00201 | 0.00099 | 0.00113 |
| *Eubacterium_G* | 0.00195 | 0.00173 | 0.00183 | 0.00198 | 0.00116 | 0.00142 |
| *Eubacterium_I* | 0.00206 | 0.00189 | 0.00210 | 0.00195 | 0.00202 | 0.00196 |
| *Eubacterium_J* | 0.00133 | 0.00159 | 0.00213 | 0.00168 | 0.00166 | 0.00121 |
| *Eubacterium_R* | 0.00279 | 0.00305 | 0.00454 | 0.00406 | 0.00383 | 0.00356 |
| *Evtepia* | 0.00142 | 0.00115 | 0.00172 | 0.00129 | 0.00128 | 0.00137 |
| *Faecalibacillus* | 0.01967 | 0.02160 | 0.02892 | 0.01659 | 0.02535 | 0.02661 |
| *Faecalibacterium* | 0.04842 | 0.06674 | 0.06300 | 0.07385 | 0.05837 | 0.05483 |
| *Faecalimonas* | 0.00131 | 0.00108 | 0.00377 | 0.00301 | 0.00196 | 0.00113 |
| *Faecousia* | 0.00143 | 0.00146 | 0.00177 | 0.00190 | 0.00228 | 0.00243 |
| *Fimenecus* | 0.00196 | 0.00386 | 0.00156 | 0.00190 | 0.00347 | 0.00151 |
| *Fusicatenibacter* | 0.02592 | 0.02711 | 0.03019 | 0.02427 | 0.03114 | 0.02295 |
| *Gemmiger_A_73129* | 0.03608 | 0.04933 | 0.04605 | 0.04178 | 0.04386 | 0.03894 |
| *Gordonibacter* | 6.93e-05 | 9.39e-05 | 7.72e-05 | 3.53e-05 | 9.26e-05 | 3.96e-05 |
| *Haemophilus_D_735815* | 0.00033 | 0.00096 | 0.00016 | 0.00028 | 0.00092 | 0.00051 |
| *Holdemanella* | 0.00900 | 0.01197 | 0.00841 | 0.00719 | 0.00747 | 0.00539 |
| *Holdemania* | 7.36e-05 | 8.86e-05 | 6.43e-05 | 9.06e-05 | 7.68e-05 | 6.78e-05 |
| *Howardella* | 0.00036 | 0.00030 | 0.00028 | 0.00025 | 0.00018 | 0.00028 |
| *Hungatella_A_128155* | 0.00040 | 0.00019 | 9.52e-05 | 7.76e-05 | 4.59e-05 | 3.83e-05 |
| *Hydrogeniiclostridium* | 5.38e-05 | 2.51e-05 | 3.14e-05 | 3.78e-05 | 3.13e-05 | 1.62e-05 |
| *Intestinibacter* | 0.00592 | 0.00472 | 0.00540 | 0.00398 | 0.00363 | 0.00515 |
| *Lachnoclostridium_B* | 0.00090 | 0.00116 | 0.00132 | 0.00102 | 0.00109 | 0.00117 |
| *Lachnospira* | 0.00640 | 0.00729 | 0.00669 | 0.00821 | 0.00754 | 0.00497 |
| *Lactococcus_A_346120* | 0.00017 | 0.00039 | 0.00127 | 0.00049 | 0.00064 | 0.00135 |
| *Lactonifactor* | 9.56e-05 | 9.05e-05 | 4.75e-05 | 5.32e-05 | 9.17e-05 | 0.00010 |
| *Lawsonibacter* | 0.00182 | 0.00173 | 0.00188 | 0.00174 | 0.00153 | 0.00131 |
| *Limiplasma* | 0.00030 | 0.00014 | 0.00030 | 0.00021 | 0.00049 | 0.00058 |
| *Limivicinus* | 0.00061 | 0.00048 | 0.00054 | 0.00121 | 0.00085 | 0.00074 |
| *Limivivens* | 0.00182 | 0.00176 | 0.00156 | 0.00179 | 0.00168 | 0.00162 |
| *Longicatena* | 0.00053 | 0.00032 | 0.00028 | 0.00029 | 0.00018 | 0.00031 |
| *Marvinbryantia* | 0.00040 | 0.00030 | 0.00027 | 0.00048 | 0.00024 | 0.00026 |
| *Massilistercora* | 0.00081 | 0.00039 | 0.00042 | 0.00033 | 0.00029 | 0.00054 |
| *Mediterraneibacter_A_155507* | 0.01919 | 0.01750 | 0.01760 | 0.01453 | 0.01679 | 0.01442 |
| *Mediterraneibacter_A_155590* | 0.00027 | 0.00021 | 0.00021 | 0.00021 | 0.00019 | 0.00026 |
| *Merdicola* | 0.00183 | 0.00202 | 0.00177 | 0.00182 | 0.00147 | 0.00234 |
| *Merdisoma* | 0.00014 | 0.00010 | 0.00011 | 7.48e-05 | 8.78e-05 | 9.61e-05 |
| *Mesosutterella* | 0.00181 | 0.00248 | 0.00199 | 0.00174 | 0.00220 | 0.00193 |
| *Monoglobus* | 0.00075 | 0.00085 | 0.00098 | 0.00067 | 0.00086 | 0.00069 |
| *Muricomes_149725* | 0.00036 | 7.10e-05 | 7.01e-05 | 7.43e-05 | 0.00017 | 7.91e-05 |
| *Negativibacillus* | 0.00058 | 0.00056 | 0.00043 | 0.00050 | 0.00051 | 0.00039 |
| *Odoribacter_865974* | 0.00064 | 0.00025 | 0.00042 | 0.00028 | 0.00030 | 0.00041 |
| *Oliverpabstia* | 0.00882 | 0.01321 | 0.01085 | 0.00682 | 0.00885 | 0.00923 |
| *Onthenecus* | 0.00281 | 0.00313 | 0.00395 | 0.00261 | 0.00325 | 0.00309 |
| *Onthomonas* | 0.00040 | 0.00031 | 0.00035 | 0.00035 | 0.00055 | 0.00057 |
| *Parabacteroides_B_862066* | 0.00674 | 0.00536 | 0.00598 | 0.00761 | 0.00498 | 0.00335 |
| *Paralachnospira* | 4.52e-05 | 5.98e-05 | 1.29e-05 | 5.55e-05 | 5.93e-05 | 5.08e-05 |
| *Paraprevotella* | 0.00149 | 0.00240 | 0.00226 | 0.00029 | 7.75e-05 | 0.00052 |
| *Parasutterella* | 0.00453 | 0.00173 | 0.00354 | 0.00326 | 0.00296 | 0.00350 |
| *Pauljensenia* | 0.00022 | 0.00024 | 0.00020 | 0.00020 | 0.00025 | 0.00023 |
| *PeH17* | 0.00032 | 0.00034 | 0.00068 | 0.00054 | 0.00088 | 0.00060 |
| *Phascolarctobacterium_A* | 0.00238 | 0.00192 | 0.00236 | 0.00289 | 0.00172 | 0.00165 |
| *Phocaeicola_A_858004* | 0.09864 | 0.05342 | 0.05356 | 0.06330 | 0.06407 | 0.05038 |
| *Phocea* | 6.83e-05 | 5.83e-05 | 8.33e-05 | 7.82e-05 | 8.43e-05 | 4.53e-05 |
| *Porcincola* | 0.00037 | 0.00039 | 0.00048 | 0.00040 | 0.00048 | 0.00034 |
| *Prevotella* | 0.03584 | 0.03860 | 0.00788 | 0.02617 | 0.03626 | 0.01585 |
| *Pygmaiobacter* | 8.51e-05 | 0.00018 | 7.63e-05 | 0.00016 | 8.79e-05 | 0.00016 |
| *Romboutsia_B* | 0.01302 | 0.02035 | 0.00983 | 0.01424 | 0.01555 | 0.01851 |
| *Roseburia* | 0.00680 | 0.00648 | 0.00661 | 0.00737 | 0.00613 | 0.00618 |
| *RUG115* | 0.00114 | 0.00096 | 0.00093 | 0.00118 | 0.00082 | 0.00083 |
| *Ruminiclostridium_E* | 0.00202 | 0.00223 | 0.00176 | 0.00197 | 0.00300 | 0.00224 |
| *Ruminococcus_B* | 0.00552 | 0.00712 | 0.00844 | 0.01018 | 0.00778 | 0.00770 |
| *Ruminococcus_C_58660* | 0.00407 | 0.00330 | 0.00412 | 0.00509 | 0.00284 | 0.00462 |
| *Ruminococcus_C_59129* | 0.00016 | 0.00021 | 0.00021 | 0.00019 | 0.00018 | 0.00050 |
| *Ruminococcus_D* | 0.00301 | 0.00232 | 0.00340 | 0.00365 | 0.00476 | 0.00414 |
| *Ruminococcus_E* | 0.02762 | 0.03138 | 0.03591 | 0.03554 | 0.02290 | 0.02623 |
| *Ruthenibacterium* | 0.00138 | 0.00092 | 0.00104 | 0.00096 | 0.00065 | 0.00095 |
| *Scatomonas* | 0.00016 | 0.00013 | 0.00020 | 0.00013 | 0.00014 | 0.00021 |
| *Schaedlerella* | 0.00121 | 0.00149 | 0.00198 | 0.00135 | 0.00095 | 0.00169 |
| *Sellimonas* | 0.00041 | 0.00021 | 0.00039 | 0.00035 | 0.00033 | 0.00059 |
| *Senegalimassilia* | 0.00025 | 0.00030 | 0.00036 | 0.00040 | 0.00040 | 0.00034 |
| *SFLA01* | 0.00011 | 7.69e-05 | 8.57e-05 | 6.30e-05 | 0.00011 | 0.00014 |
| *SFMI01* | 0.00255 | 0.00391 | 0.00330 | 0.00284 | 0.00407 | 0.00449 |
| *Slackia_A* | 7.90e-05 | 9.88e-05 | 7.21e-05 | 6.04e-05 | 7.25e-05 | 0.00024 |
| *Streptococcus* | 0.00874 | 0.01897 | 0.00873 | 0.00576 | 0.00596 | 0.01724 |
| *Terrisporobacter* | 0.00225 | 0.00237 | 0.00301 | 0.00318 | 0.00157 | 0.00208 |
| *Turicibacter* | 0.00156 | 0.00164 | 0.00219 | 0.00132 | 0.00112 | 0.00215 |
| *UBA1417* | 0.00585 | 0.00442 | 0.00517 | 0.00549 | 0.00532 | 0.00572 |
| *UBA3402* | 3.05e-05 | 0.00012 | 0.00012 | 0.00029 | 0.00022 | 0.00022 |
| *UBA644* | 2.69e-05 | 6.04e-05 | 6.42e-05 | 4.77e-05 | 8.07e-05 | 9.13e-05 |
| *UBA737* | 9.24e-05 | 0.00017 | 0.00010 | 0.00010 | 2.59e-05 | 4.91e-05 |
| *UMGS1071* | 0.00059 | 0.00041 | 0.00050 | 0.00052 | 0.00070 | 0.00055 |
| *UMGS1375* | 0.00229 | 0.00134 | 0.00165 | 0.00144 | 0.00145 | 0.00215 |
| *Unclassified Acutalibacteraceae* | 0.00092 | 0.00147 | 0.00181 | 0.00097 | 0.00123 | 0.00113 |
| *Unclassified Anaerotignaceae* | 0.00025 | 0.00023 | 0.00033 | 0.00026 | 0.00022 | 0.00023 |
| *Unclassified Anaerovoracaceae* | 0.00027 | 0.00023 | 0.00052 | 0.00040 | 0.00028 | 0.00024 |
| *Unclassified CAG-138* | 0.00042 | 0.00065 | 0.00065 | 0.00051 | 0.00108 | 0.00094 |
| *Unclassified CAG-508* | 0.00086 | 0.00037 | 0.00040 | 0.00014 | 0.00026 | 0.00043 |
| *Unclassified CAG-74* | 0.00064 | 0.00041 | 0.00043 | 0.00028 | 0.00051 | 0.00061 |
| *Unclassified Christensenellales* | 0.00014 | 7.20e-05 | 9.77e-05 | 8.18e-05 | 0.00020 | 0.00016 |
| *Unclassified Coprobacillaceae* | 0.00016 | 0.00015 | 0.00012 | 7.45e-05 | 5.88e-05 | 7.43e-05 |
| *Unclassified Enterobacteriaceae_A* | 0.00012 | 0.00054 | 0.00086 | 0.00421 | 0.00020 | 0.00039 |
| *Unclassified Enterococcaceae* | 0.00011 | 0.00060 | 4.01e-05 | 0.00016 | 0.00457 | 0.00090 |
| *Unclassified Lachnospiraceae* | 0.00460 | 0.00608 | 0.00541 | 0.00581 | 0.00561 | 0.00493 |
| *Ventrimonas* | 0.00159 | 0.00108 | 0.00114 | 0.00126 | 0.00091 | 0.00080 |
| *Vescimonas* | 0.00210 | 0.00292 | 0.00205 | 0.00295 | 0.00298 | 0.00302 |

^1^Taxa were retained if they were present in at least 10% of the samples.

**Supplemental Material: Tables**

**Table S6.** Self-reported stool consistency, as assessed by the Bristol Stool Chart.

| **Variables** | **WCM (*n* = 36)** | | **RCM+B (*n* = 36)** | | **RCM (*n* = 36)** | |
| --- | --- | --- | --- | --- | --- | --- |
|  | Pre | Post | Pre | Post | Pre | Post |
| Bristol stool score, median (IQR) | 4 (2, 5) | 4 (3, 6) | 4 (3, 5) | 4 (3.7, 5) | 4 (3, 4.25) | 4 (3, 4.25) |
| 1 - Hard lumps, % (n) | 22.2% (8) | 16.7% (6) | 16.7% (6) | 11.1% (4) | 16.7% (6) | 5.6% (2) |
| 2 - Lumpy sausage, % (n) | 11.1% (4) | 2.8% (1) | 0.0% (0) | 8.3% (3) | 0.0% (0) | 11.1% (4) |
| 3 - Sausage with cracks, % (n) | 5.6% (2) | 11.1% (4) | 13.9% (5) | 5.6% (2) | 19.4% (7) | 19.4% (7) |
| 4 - Smooth sausage, % (n) | 33.3% (12) | 25.0% (9) | 38.9% (14) | 41.7% (15) | 38.9% (14) | 38.9% (14) |
| 5 - Soft blobs, % (n) | 13.9% (5) | 16.7% (6) | 19.4% (7) | 13.9% (5) | 5.6% (2) | 16.7% (6) |
| 6 - Fluffy and mushy stool, % (n) | 8.3% (3) | 22.2% (8) | 8.3% (3) | 16.7% (6) | 13.9% (5) | 5.6% (2) |
| 7 - Watery, % (n) | 5.6% (2) | 5.6% (2) | 2.8% (1) | 2.8% (1) | 5.6% (2) | 2.8% (1) |

Change in Bristol stool scale ratings (post – pre) were not significantly different between treatments (Kruskal-Wallis test, P = 0.543)

**Supplemental Material: Tables**

**Table S7.** Gastrointestinal symptom prevalence pre- and post-intervention for the three treatment arms.

| **Variables** | **WCM (*n* = 36)** | | **RCM+B (*n* = 36)** | | **RCM (*n* = 36)** | |
| --- | --- | --- | --- | --- | --- | --- |
|  | Pre | Post | Pre | Post | Pre | Post |
| Abdominal pain, % (*n*)  None  Mild  Moderate  High  Severe | 72.2% (26)  25.0% (9)  2.8% (1)  0.0% (0)  0.0% (0) | 63.9% (23)  27.8% (10)  5.6% (2)  2.8% (1)  0.0% (0) | 77.8% (28)  19.4% (7)  0.0% (0)  2.8% (1)  0.0% (0) | 69.4% (25)  13.9% (5)  11.1% (4)  2.8% (1)  2.8% (1) | 75.0% (27)  19.4% (7)  2.8% (1)  2.8% (1)  0.0% (0) | 75.0% (27)  22.2% (8)  0.0% (0)  2.8% (1)  0.0% (0) |
| Stool frequency, % (*n*)  None  Mild  Moderate  High  Severe | 75.0% (27)  19.4% (7)  2.8% (1)  2.8% (1)  0.0% (0) | 47.2% (17)  30.6% (11)  19.4% (7)  2.8% (1)  0.0% (0) | 83.3% (30)  11.1% (4)  5.6% (2)  0.0% (0)  0.0% (0) | 50.0% (18)  30.6% (11)  11.1% (4)  5.6% (2)  2.8% (1) | 69.4% (25)  25.0% (9)  0.0% (0)  5.6% (2)  0.0% (0) | 58.3% (21)  30.6% (11)  8.3% (3)  2.8% (1)  0.0% (0) |
| Bloating, % (*n*)  None  Mild  Moderate  High  Severe | 63.9% (23)  25.0% (9)  8.3% (3)  2.8% (1)  0.0% (0) | 58.3% (21)  33.3% (12)  2.8% (1)  2.8% (1)  2.8% (1) | 83.3% (30)  5.6% (2)  11.1% (4)  0.0% (0)  0.0% (0) | 52.8% (19)  16.7% (6)  22.2% (8)  8.3% (3)  0.0% (0) | 66.7% (24)  19.4% (7)  11.1% (4)  2.8% (1)  0.0% (0) | 55.6% (20)  27.8% (10)  13.9% (5)  2.8% (1)  0.0% (0) |
| Flatulence, % (*n*)  None  Mild  Moderate  High  Severe | 55.6% (20)  38.9% (14)  2.7% (1)  2.7% (1)  0.0% (0) | 50.0% (18)  36.1% (13)  11.1% (4)  2.8% (1)  0.0% (0) | 69.4% (25)  22.2% (8)  8.3% (3)  0.0% (0)  0.0% (0) | 44.4% (16)  33.3% (12)  16.7% (6)  5.6% (2)  0.0% (0) | 61.1% (22)  30.6% (11)  8.3% (3)  0.0% (0)  0.0% (0) | 47.2% (17)  33.3% (12)  8.3% (3)  11.1% (4)  0.0% (0) |
| Fullness, % (*n*)  None  Mild  Moderate  High  Severe | 47.2% (17)  30.6% (11)  19.4% (7)  2.7% (1)  0.0% (0) | 44.4% (16)  33.3% (12)  16.7% (6)  2.8% (1)  2.8% (1) | 61.1% (22)  19.4% (7)  13.9% (5)  5.6% (2)  0.0% (0) | 38.9% (14)  38.9% (14)  13.9% (5)  8.3% (3)  0.0% (0) | 58.3% (21)  25.0% (9)  13.9% (5)  2.8% (1)  0.0% (0) | 50.0% (18)  30.6% (11)  13.9% (5)  5.6% (2)  0.0% (0) |
| Liquids, % (*n*)  None  Mild  Moderate  High  Severe | 55.6% (20)  38.9% (14)  2.7% (1)  2.7% (1)  0.0% (0) | 50.0% (18)  36.1% (13)  11.1% (4)  27.8% (1)  0.0% (0) | 69.4% (25)  22.2% (8)  8.3% (3)  0.0% (0)  0.0% (0) | 44.4% (16)  33.3% (12)  16.7% (6)  5.6% (2)  0.0% (0) | 61.1% (22)  30.6% (11)  8.3% (3)  0.0% (0)  0.0% (0) | 47.2% (17)  33.3% (12)  8.3% (3)  11.1% (4)  0.0% (0) |

No differences observed between treatments for change in self-reported gastrointestinal symptoms, including increased experience of abdominal pain, bowel movements, bloating, flatulence, fullness, and liquids (Kruskal-Wallis tests, P ≥ 0.105)

**Supplemental Material: Tables**

**Table S8.** Reported satisfaction of pita food product for the three treatment arms.^1^

| **Rating Categories** | **WCM (*n* = 36)** | **RCM+B (*n* = 36)** | **RCM (*n* = 36)** |
| --- | --- | --- | --- |
| Appearance, % (n)  Excellent  Good  Satisfactory  Needs improvement  Unsatisfactory | 18.1% (26)  47.9% (69)  26.4% (38)  6.9% (10)  0.7% (1) | 25.7% (37)  36.1% (52)  20.1% (29)  16.0% (23)  2.1% (3) | 28.9% (44)  32.9% (50)  30.3% (46)  7.9% (12)  0.0% (0) |
| Consistency/texture, % (n)  Excellent  Good  Satisfactory  Needs improvement  Unsatisfactory | 12.5% (18)  43.8% (63)  28.5% (41)  14.6% (21)  0.7% (1) | 21.5% (31)  33.3% (48)  18.8% (27)  22.9% (33)  3.5% (5) | 24.3% (35)  31.9% (46)  29.2% (42)  12.5% (18)  2.1% (3) |
| Flavor, % (n)  Excellent  Good  Satisfactory  Needs improvement  Unsatisfactory | 10.4% (15)  45.1% (65)  25.0% (36)  18.8% (27)  0.7% (1) | 18.8% (27)  31.3% (45)  24.3% (35)  24.3% (35)  1.4% (2) | 19.4% (28)  34.7% (50)  32.6% (47)  13.2% (19)  0.0% (0) |
| Overall satisfaction, % (n)  Excellent  Good  Satisfactory  Needs improvement  Unsatisfactory | 14.6% (21)  43.1% (62)  27.8% (40)  13.9% (20)  0.7% (1) | 19.4% (28)  31.3% (45)  29.9% (43)  16.0% (23)  3.5% (5) | 20.8% (30)  35.4% (51)  30.6% (44)  11.8% (17)  1.4% (2) |

^1^Reported as sum of total scores over each four-week treatment arm (total [*n* = 144] = participant [*n* = 36] x weeks [*n* = 4])

No difference in product satisfaction scores across treatments for the two food products (Kruskal-Wallis tests, *P* ≥ 0.71

**Supplemental Material: Tables**

**Table S9.** Reported satisfaction of muffin food product for the three treatment arms.^1^

| **Rating Categories** | **WCM (*n* = 36)** | **RCM+B (*n* = 36)** | **RCM (*n* = 36)** |
| --- | --- | --- | --- |
| Appearance, % (n)  Excellent  Good  Satisfactory  Needs improvement  Unsatisfactory | 33.3% (48)  47.9% (69)  13.9% (20)  4.9% (7)  0.0% (0) | 29.2% (42)  44.4% (64)  20.1% (29)  5.6% (8)  0.7% (1) | 42.4% (61)  36.8% (53)  18.8% (27)  2.1% (3)  0.0% (0) |
| Consistency/texture, % (n)  Excellent  Good  Satisfactory  Needs improvement  Unsatisfactory | 24.3% (35)  51.4% (74)  18.1% (26)  6.3% (9)  0.0% (0) | 19.4% (28)  41.0% (59)  27.1% (39)  11.8% (17)  0.7% (1) | 36.8% (53)  34.0% (49)  25.7% (37)  3.5% (5)  0.0% (0) |
| Flavor, % (n)  Excellent  Good  Satisfactory  Needs improvement  Unsatisfactory | 23.6% (34)  52.8% (76)  17.4% (25)  6.3% (9)  0.0% (0) | 26.4% (38)  38.9% (56)  22.9% (33)  8.3% (12)  3.5% (5) | 36.1% (52)  36.1% (52)  21.5% (31)  5.6% (8)  0.7% (1) |
| Overall satisfaction, % (n)  Excellent  Good  Satisfactory  Needs improvement  Unsatisfactory | 27.8% (40)  49.3% (71)  16.7% (24)  6.3% (9)  0.0% (0) | 22.9% (33)  43.1% (62)  22.2% (32)  11.1% (16)  0.7% (1) | 36.8% (53)  34.7% (50)  24.3% (35)  3.5% (5)  0.7% (1) |

^1^Reported as sum of total scores over each four-week treatment arm (total [*n* = 144] = participant [*n* = 36] x weeks [*n* = 4])

No difference in product satisfaction scores across treatments for the two food products (Kruskal-Wallis tests, *P* ≥ 0.71
